# Recent Achievements in Development of TiO_2_-Based Composite Photocatalytic Materials for Solar Driven Water Purification and Water Splitting

**DOI:** 10.3390/ma13061338

**Published:** 2020-03-15

**Authors:** Klara Perović, Francis M. dela Rosa, Marin Kovačić, Hrvoje Kušić, Urška Lavrenčič Štangar, Fernando Fresno, Dionysios D. Dionysiou, Ana Loncaric Bozic

**Affiliations:** 1 Faculty of Chemical Engineering and Technology, University of Zagreb, Marulicev trg 19, HR–10000 Zagreb, Croatia; kperovic@fkit.hr (K.P.); frosa@fkit.hr (F.M.d.R.); mkovacic@fkit.hr (M.K.); abozic@fkit.hr (A.L.B.); 2 Faculty of Chemistry and Chemical Technology, University of Ljubljana, 1000 Ljubljana, Slovenia; Urska.Lavrencic.Stangar@fkkt.uni-lj.si; 3 Photoactivated Processes Unit, IMDEA Energy, Móstoles, 28935 Madrid, Spain; fernando.fresno@imdea.org; 4 Environmental Engineering and Science Program, University of Cincinnati, Cincinnati, OH 45221–0012, USA; DIONYSDD@UCMAIL.UC.EDU

**Keywords:** TiO_2_ heterojunction, semiconductor coupling, water treatment, photocatalytic degradation, photocatalytic water splitting, H_2_ production

## Abstract

Clean water and the increased use of renewable energy are considered to be two of the main goals in the effort to achieve a sustainable living environment. The fulfillment of these goals may include the use of solar-driven photocatalytic processes that are found to be quite effective in water purification, as well as hydrogen generation. H_2_ production by water splitting and photocatalytic degradation of organic pollutants in water both rely on the formation of electron/hole (*e*^−^/*h*^+^) pairs at a semiconducting material upon its excitation by light with sufficient photon energy. Most of the photocatalytic studies involve the use of TiO_2_ and well-suited model compounds, either as sacrificial agents or pollutants. However, the wider application of this technology requires the harvesting of a broader spectrum of solar irradiation and the suppression of the recombination of photogenerated charge carriers. These limitations can be overcome by the use of different strategies, among which the focus is put on the creation of heterojunctions with another narrow bandgap semiconductor, which can provide high response in the visible light region. In this review paper, we report the most recent advances in the application of TiO_2_ based heterojunction (semiconductor-semiconductor) composites for photocatalytic water treatment and water splitting. This review article is subdivided into two major parts, namely Photocatalytic water treatment and Photocatalytic water splitting, to give a thorough examination of all achieved progress. The first part provides an overview on photocatalytic degradation mechanism principles, followed by the most recent applications for photocatalytic degradation and mineralization of contaminants of emerging concern (CEC), such as pharmaceuticals and pesticides with a critical insight into removal mechanism, while the second part focuses on fabrication of TiO_2_-based heterojunctions with carbon-based materials, transition metal oxides, transition metal chalcogenides, and multiple composites that were made of three or more semiconductor materials for photocatalytic water splitting.

## 1. Introduction

Nowadays, accessible clean water and energy resources are among the highest priorities for sustainable economic growth and societal wellbeing. Water supports life and is a crucial resource for humanity; it is also at the core of natural ecosystems and climate regulation. Water stress is primarily a water quantity issue, but it also occurs as a consequence of a deterioration of water quality and a lack of appropriate water management [1]. Environmental problems that are associated with water pollution have been a persistently important issue over recent decades, correlated negatively with the health and ecosystem. Activities of the Water JPI’s *Strategic Research and Innovation Agenda* focus on, among others, new materials and processes, energy efficiency, thus supporting key enabling technologies for clean water and wastewater treatment [2]. *EU Energy Strategies 2020*, *2030,* and *2050* set increasing standards for the reduction of greenhouse gas emissions by 20, 40, and 80–95%, respectively, which is achievable by significant investments in the development and application of new low-carbon and renewable energy technologies [3]. In light of increased energy demands and the need to reduce greenhouse gas emissions, the focus has been turned from the fossil fuels toward renewable energy resources and vectors: solar, wind, tides, waves, geothermal, biomass, biofuels, and hydrogen (H_2_) [4]. Alternative fuels are required to have as small environmental footprint, and be storable and economical, whereas H_2_ satisfies the first two conditions. The research over the last decades has been focused on fulfilling the third requirement, which triggers its production by solar energy, a largely available and intrinsically renewable energy resource, through water splitting. It should be emphasized that H_2_, as a fuel, possesses higher heat content than gasoline (per unit mass) [5].

The pioneering work of Fujishima and Honda [6] for H_2_ production by photoelectrochemical water splitting while using TiO_2_ photoanode and Pt cathode opened the potential possibilities for generating this energy vector, i.e., fuel, directly from water and solar energy. Works by Bard and Frank in 1977 [7], exhibiting photocatalytic oxidation of CN to CNO^−^, and by Ollis et al. [8], studying the photocatalytic degradation of organic contaminants in water, practically opened a new research field within new water purification technologies. H_2_ production by water splitting and photocatalytic degradation of organic pollutants in water both rely on the formation of electron/hole (*e*^-^/*h*^+^) pairs at a semiconducting material upon its excitation by light with sufficient photon energy [9,10,11,12]. These processes, which can be conducted under environmentally friendly and mild conditions, are economically viable, possessing a potential of becoming effective methods to produce clean energy and water, owing to their low-cost, long-term stability, and usage of solar energy [13].

A well-suited model catalyst for photocatalytic studies is TiO_2_. Its wide application has been promoted, due to: (i) high photocatalytic activity under the incident photon wavelength of 300 < λ <3 90 nm and (ii) multi-faceted functional properties, such as chemical and thermal stability, resistance to chemical breakdown, and attractive mechanical properties [14,15]. However, harvesting a broader spectrum of solar irradiation involves the lowering of the band gap of semiconducting material, whilst inhibiting the recombination of photogenerated charges. Strategies, including doping with non-metals, incorporation or deposition of noble metals (ions), and material engineering solutions that are based on composites formation using transition metals, carbon nanotubes, dye sensitizers, conductive polymers, graphene (oxide), and semiconducting materials, present viable solutions for set tasks [9,10,15,16]. It is of great importance to combine TiO_2_ with narrow band gap semiconductors with visible light response to obtain an effective composite for photocatalytic applications. The obtained synergistic effect between two or more semiconductors will then promote efficient charge separation, sufficient visible light response, and high photocatalytic performance. With the dramatic increase of the papers published related to these topics, a comprehensive review is desirable, providing a general overview on processes occurring while using TiO_2_-based heterojunction (semiconductor) systems for photocatalytic water purification and water splitting. Despite reviews focusing on TiO_2_–based semiconductor composites [17,18], those focusing on the removal of contaminants of emerging concern (CECs) are quite scarce. In addition, this review also summarizes TiO_2_-based nanocomposites for photocatalytic water splitting providing insight into effectiveness of a variety of materials groups representing the alternative for replacing the utilization of expensive, toxic, and non-abundant materials. The first part of the review focuses on TiO_2_–based heterojunction (semiconductor-semiconductor coupling) composites, being selected on the basis of band gap energies suitable to make heterojunctions with documented applications providing promising results in CECs treatment and stability of prepared materials, and also respecting their most recent applications for the photocatalytic degradation of CECs (i.e., demonstrating the current focus within the field), such as pharmaceuticals and pesticides, with critical insight into the pollutants removal mechanism. The second part targets the most recent achievements in the field of fabrication of TiO_2_-based heterojunctions with carbon based materials, transition metal oxides, transition metal chalcogenides, and multiple composites that were made of three or more semiconductor materials for photocatalytic water splitting.

## 2. Photocatalytic Water Treatment 

**Figure 1:**
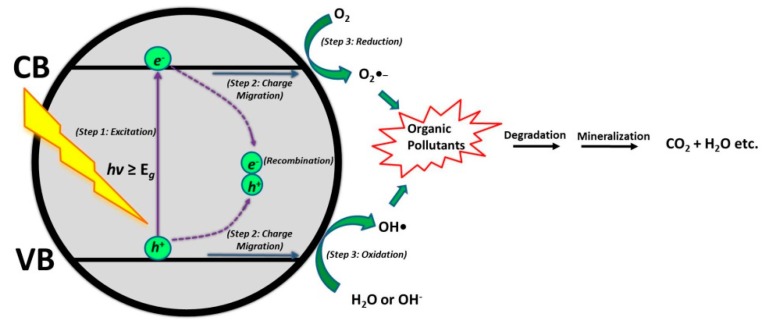
Photocatalytic reaction mechanism over semiconductor material.

The general mechanism of semiconductor photocatalysis (Figure 1) is composed of three main steps: 1. *e*^−^/*h*^+^ pairs are generated on the surface of the semiconductor under illumination with the required wavelength or energy; then, 2.) photogenerated charges (i.e., *e*^−^/*h*^+^) migrate to the surface of the semiconductor; and lastly, 3.) *e*^−^ and *h*^+^ induce redox reactions on the surface that facilitate destruction of organic pollutants [19,20]. As stressed above, TiO_2_ is still the most studied and widely used material for photocatalytic degradation reactions. However, TiO_2_ suffers from fast *e*^−^/*h*^+^ pair recombination and large band gap (*E_g_* = 3.1–3.2 eV), which can only be excited under UV light irradiation. The strategies for improving these issues are provided above, while, among them, semiconductor-coupling presents a viable structure-properties engineering solution for the enhancement of TiO_2_ photocatalytic activity due to the simultaneously reduced *e*^−^/*h*^+^ recombination rate and enhanced visible light absorption [21].

**Figure 2:**
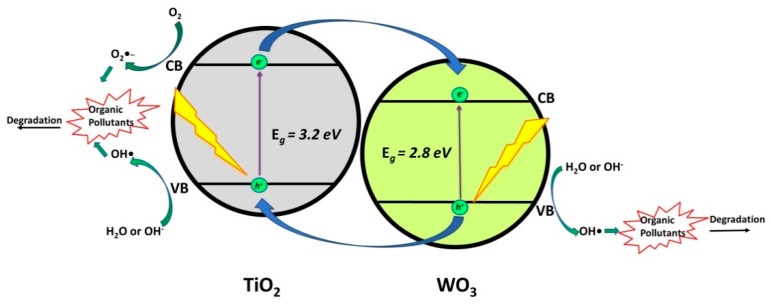
Photocatalytic degradation mechanism over TiO_2_/WO_3_ composite.

**Figure 3:**
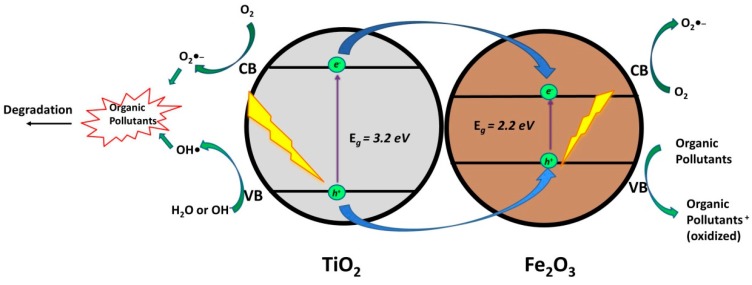
Photocatalytic degradation mechanism over TiO_2_/Fe_2_O_3_ composite.

Three main types of heterojunction architectures are reported for TiO_2_/semiconductor composites [22]. In Type I heterojunction, the conduction band (CB) of TiO_2_ is higher in energy (more negative potential) when compared to the CB of semiconductor 2 and the valence band (VB) of TiO_2_ is lower in energy (more positive potential) as compared to the VB of semiconductor 2 [23,24]. This leads to the accumulation of photogenerated *h*^+^ and *e*^−^ in semiconductor 2. In Type II heterojunction (where TiO_2_ can be semiconductor 1 or 2), the CB of semiconductor 2 is higher than the CB position of semiconductor 1 leading to facile transfer of photogenerated *e*^−^ from CB of semiconductor 2 to CB of semiconductor 1 [25]. Meanwhile, photogenerated *h*^+^ in VB of semiconductor 1 can travel to the VB of semiconductor 2, which facilitates efficient charge separation. Type III heterojunction (also known as broken gap situations) [26] shares the same charge transfer mechanism, like Type II heterojunction. In this case, the CB and VB of semiconductor 2 are higher than CB and VB of TiO_2_ [27,28]. These heterojunction types are explained in detail in the context of particular material combinations in the further text and graphically represented through Figures 2, 3 and 5–10.

### 2.1. Coupling of TiO_2_ with Metal Oxides

#### 2.1.1. TiO_2_/WO_3_

Tungsten oxide (WO_3_), which is a visible light active photocatalyst with band gap of 2.4–2.8 eV, is a promising candidate for photocatalytic applications, due to its oxidative properties, nontoxicity, low cost, and stability in acidic solutions. In addition, WO_3_ directly matches the band positions of TiO_2_ to form a heterojunction (*Type II Heterojunction*) [29,30,31,32]. Several authors studied the application of TiO_2_/WO_3_ composites for the degradation of various CECs; either pesticides or pharmaceuticals (Table 1). Hence, Macias et al. [24] studied the photocatalytic degradation of herbicide 2,4-dichlorophenoxyacetic acid (2,4-D) while using TiO_2_/WO_3_ composites under natural sunlight. They reported the rather high effectiveness of the studied system: 94.6% degradation of 2,4-D and 88.6% mineralization of overall organic content under two and four hours of natural sunlight irradiation, respectively. 

Besides, they studied the mechanisms that are responsible for forming reactive species within the system and, based on their findings, proposed that, upon forming *e*^−^/*h*^+^ pairs under solar irradiation, photogenerated *e*^−^ from CB of TiO_2_ are transferred to CB of WO_3_. Consequently, W^6+^ was first reduced to W^5+^ on WO_3_ surface, while the W^5+^ ions are then oxidized to W^6+^ by adsorbed O_2_ producing superoxide anion radical (O_2_^●‒^). The photogenerated *h*^+^ in VB of WO_3_ are transferred to VB of TiO_2_ where they reacted with water (or hydroxyl ions, HO^−^) forming hydroxyl radicals (•OH) (Figure 2). The generated reactive oxygen species (ROS) promoted the degradation of 2,4-D and its intermediates, eventually yielding rather high mineralization extents, while their occurrence in the system was confirmed through tests with common scavenging agents (e.g., tert-butanol (TB) for •OH, formic acid (FA) for *h*^+^, and *p*-benzoquinone (BQ) for O_2_^●‒^) [24].

The same composite type was used in the degradation of pharmaceuticals. Hence, Mugunthan et al. [30] treated diclofenac (DCF) while using TiO_2_/WO_3_ composites under 4 hrs of visible light irradiation and reported a maximum of 92% mineralization of overall organic content. They also elucidated the DCF degradation pathway by LC/MS measurements, which included C-N cleavage in the DCF molecule forming benzene-ring based intermediates at the first stage, and open-ring intermediates at the later stage, which were eventually mineralized. Such findings were quite similar to other studies employing •OH based processes in the degradation of DCF ([33,34]), thus implying the important role of formed ROS, primarily •OH, in the case of TiO_2_/WO_3_ solar driven photocatalysis as well. Arce-Sarria et al. [35] studied the performance of TiO_2_/WO_3_ composite for the degradation of another pharmaceutical, Amoxicillin (AMX), in pilot scale reactor, where they achieved 64.4% degradation.

Besides “pure” TiO_2_/WO_3_ composite, several authors studied the performance of its enriched analogues (Table 1). Hence, Cordero-García et al. [32] studied DCF degradation by WO_3_/C-doped TiO_2_ and reported 100% DCF degradation and 82.4% mineralization of the overall organic content under 250 kJ/m^2^ and 400 kJ/m^2^ of solar-accumulated energy, respectively. They also stated that the WO_3_/C-doped TiO_2_ composite showed superior photocatalytic activity for the complete degradation and mineralization of DCF when comparing to the pristine TiO_2_, used as benchmark material. Based on the findings on elucidated mechanisms within the studied composite and DCF degradation pathway provided, the authors concluded that the incorporation of elemental carbon to TiO_2_ crystal structure promoted the formation of a C2p-hybridized valence band that lowered the band gap of TiO_2_ by mixing with O2p orbitals. As a result, upon visible light irradiation, TiO_2_ generates *e*^−^/*h*^+^ pairs, where the photogenerated *e*^−^ are promoted to the Ti 3d states (VB), thus reducing Ti^4+^ to Ti^3+^. Ti^3+^ can be easily oxidized by WO_3_ due to the differences in the reduction potential between TiO_2_ (−0.70 V vs NHE) and WO_3_ (−0.03 V vs NHE). Subsequently, W^6+^ traps photogenerated *e*^−^ to form its reduced state W^5+^, while the redox reaction occurs further by returning to its original oxidation state in reaction with adsorbed O_2_ on the composite catalyst surface (similarly as discussed above in the case of “pure” TiO_2_/WO_3_), thus leading to improved charge separation and the formation of ROS, which contributed in DCF degradation and mineralization of formed intermediates. The same authors studied the degradation of DCF with another enhanced WO_3_/TiO_2_ composite (N-doped TiO_2_), and again reported high degradation and mineralization rates; 100% according to both indicators under 250 kJ/m^2^ and 400 kJ/m^2^ of solar-accumulated energy, respectively [31]. They stressed that the same mechanism that was responsible for the enhancement of photocatalyst activity in C-doped WO_3_/TiO_2_ composite [32] also improved the performance of N-doped WO_3_/TiO_2_ [31]. 

#### 2.1.2. TiO_2_/Fe_2_O_3_

Iron oxide (α-Fe_2_O_3_) is a promising candidate for photocatalytic applications, due to its abundance, nontoxicity, low cost, stability in aqueous solutions (pH > 3), and narrow band gap (2.0–2.2 eV), which directly matches the band positions of TiO_2_ to form heterojunction (*Type I Heterojunction*) [23,36].

Several authors report the photocatalytic degradation of CECs using TiO_2_/Fe_2_O_3_ composites (Table 2). Hence, Mirmasoomi et al. [37] used TiO_2_/Fe_2_O_3_ as a catalyst for photocatalytic degradation of Diazinon (DZ). The authors reported an optimized system with maximum degradation of DZ equal to 95.07% within 45 min. under visible light irradiation. In another study by Moniz et al. [23], photocatalytic degradation of 2,4-D while using TiO_2_/Fe_2_O_3_ composites was investigated, reporting 100% 2,4-D degradation and 100% mineralization of overall organic content within 2 h and 3 h, respectively, using irradiation from a 300 W Xenon Lamp. The authors found out that, when compared to the benchmark TiO_2_ (P25), the TiO_2_/Fe_2_O_3_ composite shows superior photocatalytic activity. Based on photoluminescence and photocurrent studies, the TiO_2_/Fe_2_O_3_ composite exhibits enhanced separation of *e*^−^/*h*^+^ pairs due to the formed heterojunction. The proposed mechanism was supported with DFT studies, which firstly involved the transfer of photogenerated *e*^−^ from TiO_2_ CB to Fe_2_O_3_ CB. In addition, Fe_2_O_3_ binds strongly with (dissolved) oxygen, thus aiding the photoelectron transfer. This *in-situ* second stage mechanism facilitates the facile migration of *h*^+^ from the VB of TiO_2_ [23]. Macías et al. [24] studied the same system, TiO_2_/Fe_2_O_3_ composites for photocatalytic degradation of 2,4-D, but while using natural sunlight. The authors reported 96.8% 2,4-D degradation and 90.0% mineralization of overall organic content under two hours and four hours, respectively. Contrary to the presented mechanism of Moniz et al. [23], Macias et al. [24] proposed that the incorporation of Fe_2_O_3_ causes the photogenerated *e*^−^ in CB of TiO_2_ to be transferred to CB of Fe_2_O_3_, promoting the reduction of Fe^3+^ to Fe^2+^. Photogenerated *h*^+^ in VB of TiO_2_ are transferred to VB of Fe_2_O_3_, which leads to the regeneration of Fe^3+^ and avoids the recombination of *e*^−^/*h*^+^ pairs at TiO_2_ surface. In addition, Fe_2_O_3_ (*E*_g_ = 2.2 eV) can be excited by visible light irradiation producing photogenerated *e*^−^/*h*^+^ pairs. Photogenerated *e*^−^ in CB of Fe_2_O_3_ can be transferred to O_2_ dissolved in water to form O_2_^●‒^, while photogenerated *h*^+^ in VB of Fe_2_O_3_ can facilitate generation of •OH eventually contributing to the degradation of present organics [24] (Figure 3). The formation of mentioned ROS and their involvement in degradation of targeted pollutant was confirmed through common scavenging tests using TB, FA, and BQ.

The photocatalytic degradation of the pharmaceutical tetracycline (TC) and its derivatives, such as oxytetracycline (OTC), using TiO_2_/Fe_2_O_3_ materials has also been reported. Hence, it was found out that, under visible light irradiation (λ = 400–750 nm), α-Fe_2_O_3_ was activated and generated *e*^−^/*h*^+^ pairs, and then photogenerated *e*^−^ from CB of α-Fe_2_O_3_ moved to TiO_2_ trapping sites for atmospheric O_2_ to form O_2_^●‒^, which was proven to largely contribute to the degradation of OTC. On the other hand, the photogenerated *h*^+^ from VB of α-Fe_2_O_3_ primarily reacted with OH^−^, resulting in the generation of •OH, which also contributed to the degradation of OTC. When compared to TiO_2_ reference material, the TiO_2_/Fe_2_O_3_ composite exhibited an enhanced photocatalytic activity under visible light due to efficient *e*^−^/*h*^+^ separation, as stated above [38]. The same authors [38,39] also studied the degradation mechanism of OTC while using LC/MS TOF analysis and, based on the formed intermediates, established the OTC degradation pathway, and concluded that •OH mainly mediated degradation. Besides, “pure” TiO_2_/Fe_2_O_3_, enriched analogue with carbon nanotubes (CNTs) was also studied (Table 2). Hence, TiO_2_/Fe_2_O_3_/CNTs was used as the catalyst for photocatalytic degradation of TC**,** under visible light illumination [40]. It was found that the effectiveness of photocatalytic degradation of TC within 90 min. treatment using TiO_2_/Fe_2_O_3_/CNTs was almost twice higher when comparing to that achieved by benchmark TiO_2_; 89.41% and 47.64%, respectively. The authors attributed the improved photocatalytic efficiency to the presence of the CNT, which acted as a photogenerated *e*^−^ acceptor, thereby suppressing *e*^−^/*h*^+^ recombination [40]. In another study, the core-shell structured α-Fe_2_O_3_ (with TiO_2_ shell of around 15 nm) exhibited 100% TC removal in 90 min. [41]. The degradation improvement was ascribed to the addition of H_2_O_2_ in the system, which generated more ROS than by the common photocatalytic mechanisms described above [41]. Hence, the contribution of H_2_O_2_ in such a system can be described through restraining *e*^−^/*h*^+^ recombination and increasing HO• generation in the system, as in Equation (1) [15]:

(1) $$H_{2}O_{2}+H^{+}+e^{-}\to \mathrm{HO}•+H_{2}O$$

#### 2.1.3. TiO_2_/Spinel Ferrite

Spinel ferrites (MFe_2_O_4_) are metal oxides, where M is a divalent ion (i.e., Mg^2+^, Ca^2+^, Sr^2+^, Ni^2+^, Zn^2+^, etc.), serving as promising candidates for photocatalytic applications due to their narrow band gap range (1.3–2.2 eV) and magnetic properties [42,43]. Spinel ferrites band positions match TiO_2_, thus possessing compatibility to form a heterojunction (*Type II Heterojunction*) [44,45,46,47].

**Figure 4:**
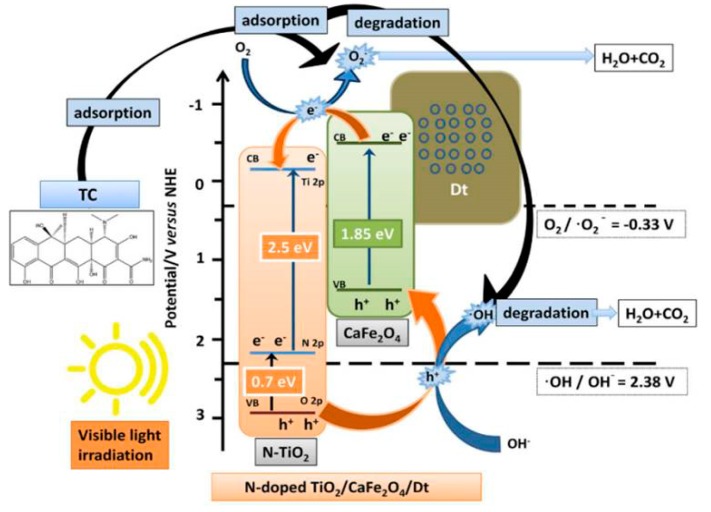
Proposed mechanism for the tetracycline (TC) photodegradation process using N-doped TiO_2_/CaFe_2_O_4_/ diatomite [44].

The literature provides applications of MFe_2_O_4_/TiO_2_ materials as photocatalysts in treatment of CECs, as in the case of previously discussed TiO_2_-based composites, however, it should be noted that authors within such composites used modified TiO_2_ (Table 3). Hence, Chen et al. [44] studied photocatalytic degradation of TC and its derivatives using N-doped TiO_2_/CaFe_2_O_4_/diatomite, and reported 91.7% removal of TC within 150 min. under visible light irradiation. The authors studied the composite stability and reusability; the results obtained after five cycles indicates that employed composite is rather stable, enabling 89.2% removal of TC. They also proposed the photocatalytic mechanism occurring within the composite; the excitation of both N-TiO_2_ and CaFe_2_O_4_ by visible light leads to the formation of *e*^−^/*h*^+^ pairs (Figure 4). The photogenerated *e*^−^ in CB of N-TiO_2_ can directly react to adsorbed O_2_ generating O_2_^●‒^, while photogenerated *h*^+^ in VB of N-TiO_2_ directly react with H_2_O and OH^−^ producing •OH. Simultaneously, photogenerated *e*^−^ in CB of CaFe_2_O_4_ can undergo the same mechanism (i.e., reaction with O_2_ to produce O_2_^●‒^). In addition, the formed heterojunction helps the migration of *e*^−^ from CB of CaFe_2_O_4_ to CB of N-TiO_2_, and *h*^+^ from VB of N-TiO_2_ to VB of CaFe_2_O_4_ (Figure 4). Such a transfer of charge carriers between the two semiconductors hinders the recombination process and enhances the photocatalytic activity of the composite, thus leading to more efficient generation of ROS (O_2_^●‒^ and •OH) [44]; the existence of formed ROS was confirmed through scavenging tests while using isopropyl alcohol (IPA), ammonium oxalate (AO), and BQ for •OH, *h*^+^ and O_2_^●‒^, respectively. Such behavior is confirmed by studying the degradation pathway of TC; it was found that the TC intermediates match those that formed through radical driven reactions undergoing in the first step demethylation and hydroxylation. The second step considered the removal of functional groups (amino, hydroxyl, and methyl) and further ring opening reactions that are mainly mediated by photogenerated *h*^+^, yielding small fragments that were eventually mineralized [44]. Such a pathway confirmed the dual role of photogenerated *h*^+^, as a promotor •OH generation and as sites for the direct oxidation of adsorbed organics. There are several other studies investigating the application of different MFe_2_O_4_/TiO_2_ materials (N-doped TiO_2_/SrFe_2_O_4_ diatomite [46]; Ce/N-co-doped TiO_2_/NiFe_2_O_4_/ diatomite and ZnFe_2_O_4_/TiO_2_ [47]) for the photocatalytic degradation of CECs, such as TC, OTC, and bisphenol A (BPA). Interestingly, the same mechanisms responsible for charge transfer and consequent generation of ROS were reported, regardless of the different M type within the spinel ferrite part of composite and/or TiO_2_ (non-doped or doped with metal and/or non-metal ions). 

#### 2.1.4. TiO_2_/Cu_2_O

**Figure 5:**
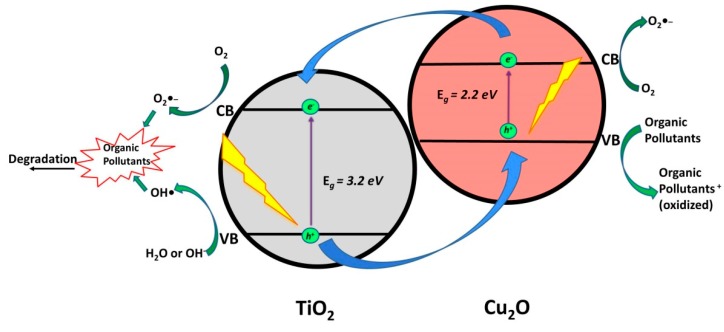
Photocatalytic degradation mechanism over TiO_2_/Cu_2_O composite.

Cu_2_O, a *p*-type semiconductor (*E*_g_ = 2.0–2.2 eV), is also a good candidate for making heterojunctions with TiO_2_ (*Type II Heterojunction*). Hence, the photocatalytic degradation of various CECs (TC [48], and tetrabromodiphenyl ethers [49]) using TiO_2_/Cu_2_O composite materials was reported under solar light irradiation (Table 4). Based on the findings in the mentioned studies, the photocatalytic mechanism of TiO_2_/Cu_2_O under solar light illumination involves the activation of both Cu_2_O and TiO_2_ to generate *e*^−^/*h*^+^ pairs (Figure 5). Photogenerated *e*^−^ in CB of Cu_2_O then can migrate to CB of TiO_2_ and, along with photogenerated *e*^−^ in CB of TiO_2_, react with O_2_ to form O_2_•^_^. Simultaneously, photogenerated *h*^+^ in VB of Cu_2_O can be directly involved in the oxidation of adsorbed organics, while photogenerated *h*^+^ in VB of TiO_2_ can directly oxidize adsorbed organics or react with H_2_O (i.e., OH^−^) and generate •OH. Besides, these *h*^+^ can also directly migrate to the VB of Cu_2_O, thus leading to effective charge separation that improves the overall photocatalytic activity of the composite [48].

#### 2.1.5. TiO_2_/Bi_2_O_3_

**Figure 6:**
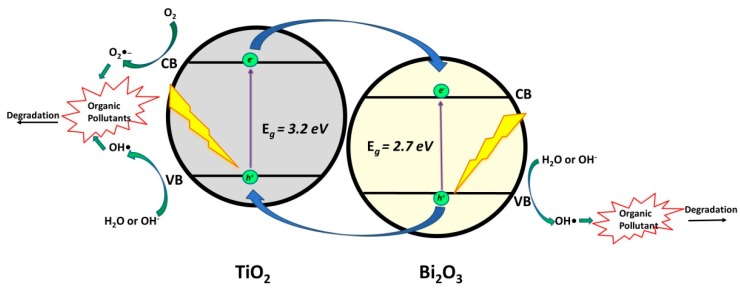
Photocatalytic degradation mechanism over TiO_2_/Bi_2_O_3_ composite.

Bi_2_O_3_, a semiconductor with band gap range in the visible region (2.5–2.8 eV), is also a good candidate for making heterojunctions with TiO_2_ (*Type II Heterojunction*). Studies including its application in photocatalytic degradation of CECs (quinalphos [50] and ofloxacin [51]) under solar light irradiation (Table 5) revealed the occurring photocatalytic mechanism. Both of the composite phases can be activated under solar irradiation generating *e*^−^/*h*^+^ pairs (Figure 6). Accordingly, photogenerated *h*^+^ in VB of TiO_2_ are involved in the production of •OH (through reactions with H_2_O, i.e., OH^−^) as of *e*^−^/*h*^+^ pairs. In addition, *h*^+^ in VB of Bi_2_O_3_ can be transferred to VB of TiO_2_ that contributes to the direct oxidation of adsorbed organics or the generation of •OH [51]. 

### 2.2. Coupling of TiO_2_ with Metal Sulfides

**Figure 7:**
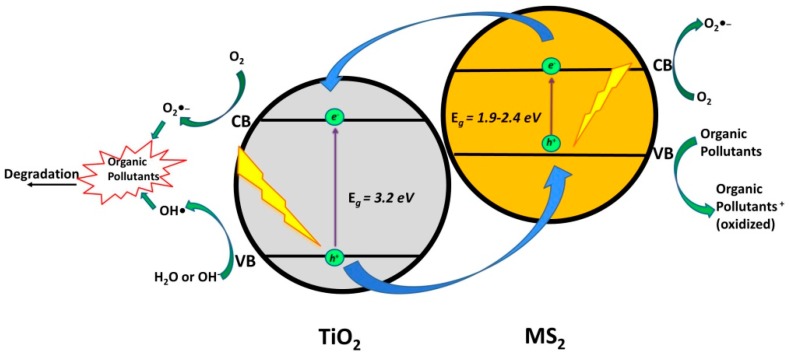
General photocatalytic degradation mechanism over TiO_2_/MS (M = Cd or Cu) or MS_2_ (M = Mo and Sn) composite.

Cadmium sulfide (CdS), a metal sulfide semiconductor with a visible light range band gap (*E*_g_ = 2.1–2.4 eV), has been proven to be compatible with TiO_2_, due to its higher position of CB than that of TiO_2_ (*Type II Heterojunction*) (Figure 7) [25,52]. However, one should be aware that its application can lead to adverse effects due to its instability, resulting in the leaching of toxic Cd^2+^ during treatment [53]. Although its CB and VB positions are thermodynamically favorable for photocatalytic application, CdS as a photocatalytic material faces serious problems. Next to the above-mentioned promotion of toxic effects, issues, like poor stability due to photocorrosion and limited separation efficiency of photogenerated charge carriers, do not speak in favor of CdS application [54,55]. Photocorrosion is not only related to the photogenerated *h*^+^ in semiconductor itself that oxidizes S^2–^ and release Cd^2+^ to the solution, but also with newly formed O_2_, where higher solubility in water leads to more dramatic levels of photocorrosion of CdS [54,56]. However, CdS was widely investigated in photocatalytic purposes, even in recent studies that focused on the degradation of CECs (ofloxacin, ciprofloxacin, tetracycline, and 17α-ethynylestradiol), where it was used in various forms (nano-rods, nano-belts) [25,52,57,58] (Table 6). Generally, upon visible light illumination, CdS is excited and generates the *e*^−^/*h*^+^ pair, where photogenerated *e*^−^ in CB of CdS migrates to CB of TiO_2_ and is consumed in reactions with O_2_ to produce O_2_^●‒^, while *h*^+^ remain in the VB of CdS. 

Copper sulfide (CuS), which is another metal sulfide semiconductor with narrow band gap of 2.0 eV, has also been reported to be coupled with TiO_2_ (*Type II Heterojunction*) [59,60]. Jiang et al. [59] reported a 85.5% degradation of enrofloxacin and 27.7% mineralization of overall organic content using immobilized CuS/TiO_2_ nanobelts (Table 6). They elucidated the mechanisms occurring in the composite upon excitation by solar irradiation. Hence, such broad wavelengths excited both composite phases (CuS and TiO_2_) and resulted in *e*^−^/*h*^+^ pairs, while the transfer of charges was analogous, as in the case of the CdS/TiO_2_ composite. Photogenerated *e*^−^ in CB (−0.33 eV) of CuS underwent transfer to CB (−0.19 eV) of TiO_2_ and were consumed in reactions with O_2_ forming O_2_^●‒^. Photogenerated *h*^+^ in VB of CuS remained there and present potential active sites for direct degradation of organics that were adsorbed at the CuS surface, since they cannot be involved in generation of •OH due to too high energy band positioning. On the other hand, photogenerated *h*^+^ in VB of TiO_2_ can directly react with adsorbed organics and OH^−^ generating •OH. Chen et.al [60], incorporated Au nanoparticles to CuS/TiO_2_ nanobelts structure to enhance the photocatalytic degradation ability of the composite by capturing *e*^−^ and, consequently, suppressing the recombination of photogenerated charges. As a result, they obtained 96% degradation of OTC and 68% mineralization of the overall organic content within one hour under artificial sunlight illumination. Accordingly, the mechanism of such enriched CuS/TiO_2_ composite involves, besides the above discussed mechanism, the path considering the transfer of *e*^−^ to Au, which leads to enhanced charge separation, thus delaying recombination. In such a case scenario, photogenerated *h*^+^ would have higher probability to react either with adsorbed organics or with HO^‒^ in order to generate •OH (exclusively those in VB of TiO_2_), thus contributing to the overall system efficiency. The involvement of formed ROS into reaction mechanisms for OTC degradation was confirmed by scavenging tests using TB, AO, and BQ.

Molybdenum disulfide (MoS_2_), a two-dimensional (2D) layered metal chalcogenide with an indirect band gap of 1.1 eV and 1.9 eV direct band gap in monolayered form, with unique structure, low-cost, high thermal stability, and electrostatic integrity, is also a suitable candidate for forming heterojunction with TiO_2_ (*Type II Heterojunction*) [61,62,63]. Hence, Kumar et al. [64] reported its application in the photocatalytic degradation of paracetamol. Furthermore, Irandost et al. [61] applied the modified MoS_2_/TiO_2_ composite (they used N,S-co-doped TiO_2_) in the photocatalytic degradation of DCF under visible LED lamp irradiation (Table 6). Hence, the synergistic effect of dopants in TiO_2_ promoted its visible light activity, yielding the formation of *e*^−^/*h*^+^ pairs in both composite phases. The mechanism of charge formation and consequent transfer was similar, as described above for CuS/TiO_2_, which was excited by solar irradiation. Hence, photogenerated *e*^−^ in CB of N,S-co doped TiO_2_ and CB of MoS_2_ were able to undergo reactions with O_2_ forming O_2_^●‒^, while *h*^+^ in VB of TiO_2_ promoted •OH formation in reactions with HO^‒^ and provide the direct oxidation of adsorbed organics, while, again, *h*^+^ in MoS_2_ were able to do only the latter. The importance of •OH and *h*^+^ in DCF degradation was confirmed by trapping agents used in scavenging tests: TB and potassium iodide (KI), respectively.

Tin sulfide (SnS_2_), which is a metal sulfide semiconductor with band gap of 2.2 eV [65], has also been reported to be coupled with TiO_2_ (*Type II Heterojunction*) [66,67]. Hence, Kovačić et al. [66] reported improved the degradation of 17β-estradiol (E2), for 51%, using SnS_2_/TiO_2_ when comparing to the benchmark material (P25) TiO_2_ under solar irradiation. A similar improvement was obtained by comparing performances of the same materials in the case of DCF degradation [67] (Table 6). The reason for such improvement relies on the potential of photogenerated *e*^−^ in CB of SnS_2_ to migrate to CB of TiO_2_, while *h*^+^ remained at the VB of SnS_2_. In such case, the efficient separation of charges is achieved, thus facilitating the improved redox reactions, enabling effective degradation of adsorbed organics directly on the surface by *h*^+^, in spite of the limited ability of such a composite to generate •OH. Accordingly, the adsorption has been shown as an important step in the effectiveness of the SnS_2_/TiO_2_ composite. Kovačić et al. [67] utilized DFT calculations to study the surface interaction of polar compounds (DCF) and non-polar compounds (memantine) at SnS_2_/TiO_2_ composite and found that DCF was more efficiently degraded due to much higher adsorption ability in comparison to memantine, which is one of its structure feature limitations (amine functionality).

### 2.3. Coupling of TiO_2_ with Silver- Based Semiconductors

Silver Phosphate (Ag_3_PO_4_), a promising semiconductor with narrow band gap (*E*_g_ ≥ 2.4 eV), showed good photocatalytic performance in the degradation of organic pollutants under visible light irradiation [68,69]. Namely, Ag_3_PO_4_ exhibits a quantum efficiency of up to 90% [68] and it can absorb wavelengths that are shorter than ~530 nm [69]. Despite the qualities of Ag_3_PO_4_ as a potential photocatalyst, it still suffers from limitations, such as photocorrosion, small but not negligible solubility in water (K_sp_ = 1.6 × 10^−16^), and particle agglomeration upon synthesis [70]. To overcome these limitations, constructing a heterojunction between Ag_3_PO_4_ and a compatible semiconductor has attracted attention due to the increase in charge separation and production of more ROS [71]. The positions of VB and CB in TiO_2_ directly match the Ag_3_PO_4_ band positions, thus providing the compatibility to form a heterojunction.

Hence, Wang et al. [72] investigated the performance of TiO_2_ nanotubes/Ag_3_PO_4_ quantum dots for the degradation of TC under visible light illumination, and reported a high removal rate within a short treatment period; 90% TC removal within 8 min (Table 7). 

**Figure 8:**
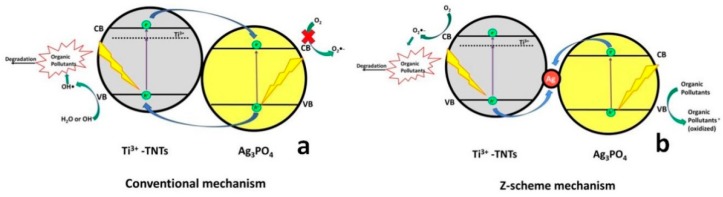
Photocatalytic mechanisms Ti^3+^-TNTs/Ag_3_PO_4_ (**a**) conventional heterojunction, and (**b**) Z-scheme heterojunction.

**Figure 9:**
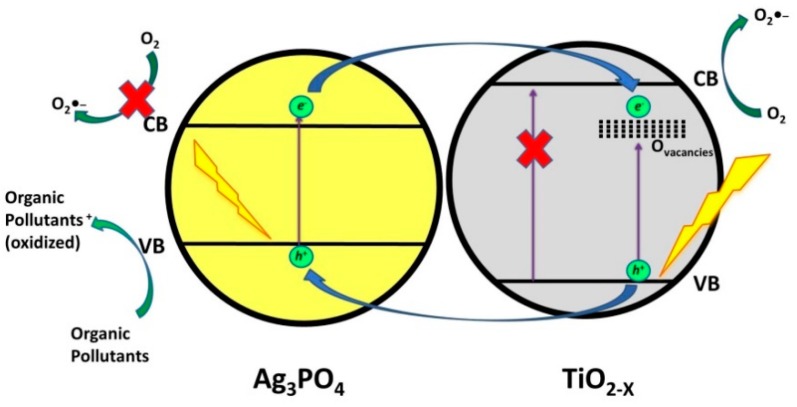
Photocatalytic reaction mechanism of TiO_2−X_/Ag_3_PO_4_ under visible light irradiation.

The conventional heterojunction transfer mechanism (Figure 8a) explains that the photogenerated *h*^+^ in the composite would be promoted from the VB of Ag_3_PO_4_ to VB of Ti^3+^-doped TiO_2_ nanotubes, where can react with H_2_O or HO^−^ forming •OH. Simultaneously, photogenerated e^−^ from the Ti^3+^-doped TiO_2_ nanotubes CB can react with O_2_ forming O_2_^●‒^ or may transfer to the CB of Ag_3_PO_4_. However, O_2_^●‒^ are not formed in Ag_3_PO_4_, due to the fact that the position of its CB is lower than the standard reduction potential of O_2_^●‒^/O_2_. Wang et al. [72] concluded that TC was primarily degraded by O_2_^●−^ and photogenerated *h*^+^ based on the results of the conducted electron trapping experiments. Accordingly, they have extended the study by proposing a Z-scheme heterojunction transfer mechanism (Figure 8b). Under this mechanism, Ag(0) acts a recombination center, “collecting” photogenerated *e*^−^ from CB of Ag_3_PO_4_, where they undergo recombination with the photogenerated *h*^+^ from VB of Ti^3+^-doped TiO_2_ nanotubes. In such case, photogenerated *h*^+^ on VB of Ag_3_PO_4_ might participate in the direct oxidation reactions with adsorbed organics, while the photogenerated *e*^−^ in the CB of Ti^3+^-doped TiO_2_ nanotubes can be involved in forming desired ROS, O_2_^●‒^, thus contributing to the enhanced performance of composite photocatalyst. Du et al. [73] applied analogue TiO_2_/Ag_3_PO_4_ composite employing TiO_2_ nanotube arrays for the degradation of ciprofloxacin (CIP) under solar irradiation and reported that 85.3% removal of CIP within 60 min. was facilitated through the above-mentioned mechanisms. Furthermore, Liu et al. [74] reported 95% degradation of BPA in 16 min. using TiO_2−X_/Ag_3_PO_4_ under visible light irradiation (Table 7). They reported that both composite phases, TiO_2−X_ and Ag_3_PO_4_, were excited and generated *e*^−^/*h*^+^ pairs. Hence, photogenerated *h*^+^ in VB of TiO_2−X_ are promoted to VB of Ag_3_PO_4_ and contributed to the direct oxidation of adsorbed organics, similarly as reported in the study by Wang et al. [72]. Photogenerated *e*^−^ from the CB of Ag_3_PO_4_ are transferred to oxygen vacancies (V_o_) of TiO_2_ and contributed in reactions with adsorbed O_2_ generating O_2_^●‒^ (Figure 9). They also investigated the role of these species in the degradation of BPA and found, based on monitoring BPA degradation pathway by LC/MS analysis, that intermediates are formed through two pathways: 1) hydroxylation, through reactions with O_2_^●‒^ yielding BPA-*o*-catechol; and, 2) direct oxidation by *h*^+^ forming isopropenylphenol and phenol, which was further oxidized by *h*^+^ yielding hydroquinone and its dehydrated form benzoquinone. 

**Figure 10:**
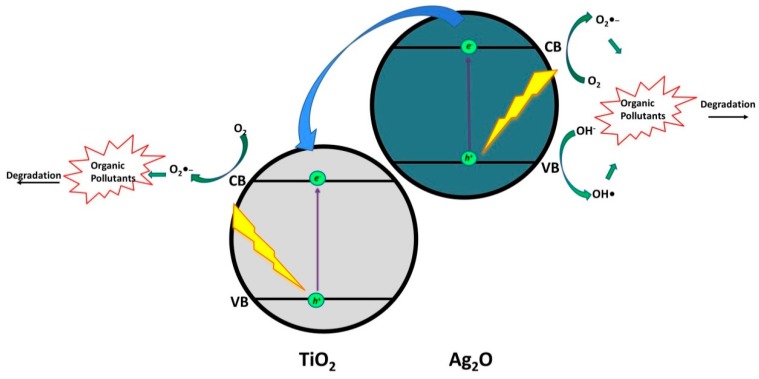
Photocatalytic degradation mechanism over TiO_2_/Ag_2_O composite.

Silver oxide (Ag_2_O), a visible light active photocatalyst with band gap of 1.2 eV, is another silver-based compound with semiconducting properties. Based on the band positions (VB and CB), it represents a promising matching candidate to form heterojunctions with TiO_2_ (*Type III Heterojunction*). Hence, photocatalytic degradation of levofloxacin (LEV) using Ag_2_O/TiO_2_ quantum dots is reported with the maximum of 81% LEV degradation within 90 min. of visible light irradiation [27]. Based on the proposed mechanism under visible light illumination (Figure 10), upon excitation of Ag_2_O, *e*^−^/*h*^+^ pairs are formed, whereas TiO_2_ is not activated due to its wide band gap. Photogenerated *e*^−^ in the CB of Ag_2_O were transferred to CB of TiO_2_ and involved in reactions with adsorbed O_2_ forming O_2_^●‒^ that participated in LEV degradation. In addition, photogenerated *h*^+^ in VB of Ag_2_O yielded the formation of •OH, through reactions with OH^−^, and participated in LEV degradation as well. The authors employed LC-MS analysis to elucidate LEV degradation pathway and, as such, establish the role of formed ROS. Hence, parent compound LEV underwent decarboxylation of the acetyl group; hydroxylation resulting in the formation of quinolone moieties; demethylation and the subsequent addition of hydrogen atom generating modifications at piperazine ring; while successive •OH attack resulted in multi-hydroxylated intermediates. Such findings confirmed the dominant role of •OH in LEV degradation. 

In another study, Gou et al. [28] investigated the application of Ag_2_O/TiO_2_/zeolite composite for solar-driven degradation of norfloxacin (NOR) (Table 7). Besides high effectiveness (98.7% NOR degradation and 83.1% mineralization of organic content within 60 min. treatment), they elucidated the NOR degradation pathway, involving in the initial stage decarboxylation, defluorination or hydroxylation of parent compound (NOR), which confirmed the involvement of both formed ROS (O_2_^●‒^ and •OH).

### 2.4. Coupling of TiO_2_ with Graphene and Graphene-Like Materials

#### 2.4.1. TiO_2_/Graphene Composites

Graphene is a zero bandgap semiconductor with a sheet-like structure (i.e., it is considered as a 2D monolayer material) consisting of sp^2^ hybridized carbon atoms with excellent thermal conductivity, optical transmittance, high mechanical strength, large surface area (2600 m^2^/g), and appreciable charge carrier transport [75]. Under light illumination, it can achieve a reverse saturation state with high density (~ 10^13^ cm^2^) of hot electrons above the Fermi level, which can be used as a powerful agent in redox reactions [76]. It was also found that the incorporation of graphene-based materials (i.e., graphene oxide and its reduced form; GO and rGO, respectively) with TiO_2_ might suppress *e*^−^/*h*^+^ pairs recombination. As such, TiO_2_/graphene-based composites were employed in the photocatalytic degradation of CECs (Table 8).

#### 2.4.2. TiO_2_/Semiconductor/Graphene Composites

As described in above sections, the coupling of TiO_2_ with other semiconductors promotes efficient charge transfer, eventually yielding improved photocatalytic activity. However, in most cases, the recombination is still an existing issue that needs to be suppressed. Such a double effect can be obtained by combining composite concept involving two semiconductors (even “pure” TiO_2_, which cannot be active under visible light) with graphene-based materials. For instance, Hao et al. [77] reported 93.2% degradation of BPA in seven hours of sunlight irradiation while using the TiO_2_/WO_3_/GO composite. The mechanism occurring in such combined composite involved the excitation of both TiO_2_ and WO_3_ under solar light irradiation (TiO_2_ utilized UV-A fraction), yielding the generation *e*^−^/*h*^+^ in both semiconductors. Hence, photogenerated *e*^−^ in CB of TiO_2_ can directly react with absorbed O_2_, producing O_2_^●−^, or it can be transferred to CB of WO_3_, and then further migrate to GO enhancing charge separation. Since the amount of adsorbed O_2_ is quite limited, the tendency of *e*^−^ to recombine with *h*^+^ is rather favored; ~90% of pairs recombine rapidly after excitation [14]. Hence, the charge separation represents an important factor in the evaluation of photocatalyst performance. Accordingly, in the case of effective separation and recombination suppression, as in the case with GO, photogenerated *h*^+^ in VB of activated composite components, e.g., of TiO_2_ and WO_3_ in the case of TiO_2_/WO_3_/GO, can be involved in a larger amount, either directly or indirectly (through formation of •OH) in the degradation of present organics. It should be noted that, in composites with two semiconductors, GO could also act as redox site, attracting photogenerated *e*^−^ and *h*^+^, thus promoting improved surface migration of charges [77]. Table 8 summarizes several works regarding TiO_2_/semiconductor/GO composites employed for the degradation of CECs with analogous mechanism, as mentioned above. 

#### 2.4.3. TiO_2_/g-C_3_N_4_

Graphitic carbon nitride (g-C_3_N_4_), a two-dimensional, metal-free polymeric π-conjugated semiconductor material, which has attracted a lot of attention [83,84,85,86,87,88,89,90,91] since the pioneering work of Wang et al. [92] in 2009, due to its high stability, visible light response with the bandgap of 2.7 eV and non-toxicity [93], thus representing a viable candidate to be applied in photocatalytic water treatment [80], has certainly been one of the most investigated photocatalysts inside carbon-based nanomaterials. It can be easily synthesized through the direct pyrolysis of nitrogen-rich precursors, such as melamine, cyanamide, dicyandiamide, and urea, but its practical application and principle drawback is low specific surface area and high rate of electron-hole recombination [83,94,95]. Therefore, g-C_3_N_4_ modification to address shortcomings are needed, e.g., as an excellent candidate to form heterojunction with TiO_2_ (*Type II Heterojunction*), due to their matched band positions (VB and CB). Hence, several studies employing g-C_3_N_4_/TiO_2_ were focused on photocatalytic degradation of CECs (Table 9). For instance, Yang et al. [96] reported 88.1% degradation of CIP within 180 min. under visible light irradiation. The authors ascribed the improved photocatalytic activity to multiple effects: (i) an increase in the surface area of the composite; (ii) good dispersity of TiO_2_ in g-C_3_N_4_ enabling the intimately coupling of composite phases; and, (iii) extension of light absorption of the composite due to low band gap of g-C_3_N_4_. Trapping experiments that were conducted revealed that photogenerated *h*^+^ were the major reactive site involved in CIP degradation.

In another study, Li et al. [97] reported the 100% degradation of Acyclovir in 90 min. using g-C_3_N_4_/TiO_2_ under visible light irradiation. However, after seven hours of continuous irradiation, any TOC removal was not noticed, implying the formation of rather recalcitrant intermediates with high resistance to degradation by ROS that formed within the studied system. Trapping experiments for formed reactive species elucidated that g-C_3_N_4_/TiO_2_ under visible light irradiation only produced *h*^+^ and O_2_^●‒^, and not the most reactive •OH, explaining limited oxidation capability and none TOC removal in the case of acyclovir degradation. This significant contribution proves that the use of g-C_3_N_4_/TiO_2_ under visible light irradiation must undergo careful laboratory tests regarding the susceptibility of targeted organics and their intermediates to degradation by *h*^+^ and O_2_^●‒^ prior to considering real scale application [97].

Several studies also showed that the tailoring of composite morphology promotes improved photocatalytic efficiency. For instance, Yu et al. [98] prepared a mesoporous g-C_3_N_4_/TiO_2_ that was applied to polysulfone ultrafiltration membranes for sulfamethoxazole (SMX) removal. It was found that mpg-C_3_N_4_/TiO_2_ exhibit 69% SMX degradation within 30 hours of sunlight irradiation. On the other hand, TiO_2_ nanosheets with exposed facets (001) (core)-g-C_3_N_4_ (shell) composite exhibit a higher degradation rate of 2.2 mg/min., which is 36% faster when compared to TiO_2_ and g-C_3_N_4_ physically-mixed composite. The improved effect is ascribed to the close interaction of TiO_2_ and g-C_3_N_4_ core-shell structure, whereas, in physically mixed composite the formed heterojunction is random and non-uniform [99].

The use of support materials, such as clays [100] and polymers [101], has been also utilized for improved adsorption capacity and the stability of g-C_3_N_4_/TiO_2_ composites. For instance, Chen et al. [101] used g-C_3_N_4_–shielding polyester fiber (PET)/TiO_2_ for photocatalytic degradation of sulfaquinoxaline and thiamethoxam. Interestingly, the composite removal efficiency for sulfaquinoxaline reached 97%, after 10 consequent cycles. Furthermore, the introduction of kaolinite with g-C_3_N_4_/TiO_2_ improved the surface area and adsorption capacity of the composite, leading to 92% degradation of CIP in 240 min. of visible light irradiation [100].

An additional approach considers doping of metals and non-metals in TiO_2_, enhancing its light absorption capacity from UV absorption to visible light absorption. Thus, incorporating doped TiO_2_ with g-C_3_N_4_ structures has also attracted great attention for the degradation of CECs. For instance, S-Ag/TiO_2_ @g-C_3_N_4_ [102] was employed for the degradation of Triclosan (TS) and yielded 92.3% degradation of TS within 60 min. under visible light irradiation. Song et al. [103] fabricated a nanofibrous Co-TiO_2_ coated with g-C_3_N_4_, which was applied to TC removal; the authors reported a consistent stability of composite photocatalyst during five consecutive cycles.

Besides doping, sensitization with dyes [104] and carbon dots [105] was also found to enhance the light absorption capacity of g-C_3_N_4_/TiO_2_ composite. For example, D35 organic dye was applied next to g-C_3_N_4_/TiO_2_ and it was found that the light absorption range was enhanced up to 675 nm [104]. On the other hand, Su et al. [105] studied the application of C dots decorated/g-C_3_N_4_/TiO_2_ for the degradation of enrofloxacin under visible light and assigned the observed enhancement to the upconversion photoluminescence properties of C dots, which convert near-infrared light wavelength into visible light wavelength [106]. As effective solutions for improving g-C_3_N_4_/TiO_2_ performance, the incorporation of graphene quantum dots [107] and another semiconductor (i.e., MoS_2_ [108], WO_3_ [109]) is also reported; such systems resulted in enhanced separation of charges and the suppression of their recombination, thus leading to improved photocatalytic activity in the degradation of CECs. 

## 3. Photocatalytic Water Splitting

**Figure 11:**
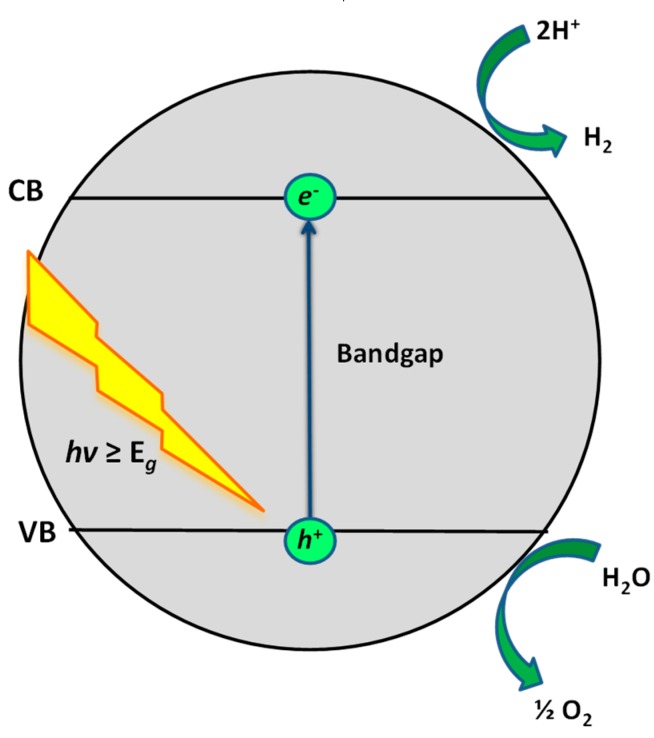
The principle photocatalytic water splitting mechanism over illuminated TiO_2_ nanoparticle.

Photocatalytic water splitting implies a non-spontaneous process, where the light photons are used to break the water molecules assisted by a photocatalyst, which generates photoexcited charge carriers, i.e., *e*^−^/*h*^+^ pairs, delivering them to the solid-liquid interface, where the redox half-reactions of water oxidation and reduction are catalyzed [110,111], analogously, as described above for photocatalytic water treatment. The difference in water splitting is that photogenerated charges (i.e., *e*^−^ and *h*^+^) need to react with H^+^ as the electron acceptor adsorbed on the photocatalyst surface or within the surrounding electrical double layer of the charged particles in order to generate H_2_ [112], instead of O_2_ generating O_2_^●−^, as in photocatalytic water treatment (Figure 1). The donors are the same; H_2_O, however, desired the product of such reaction is O_2_. Figure 11 shows the principal mechanism of photocatalytic water splitting with the use of TiO_2_ semiconductor nanoparticle. The VB and CB of semiconductor or their composites have to have favorable positions in order to enable occurrence of such reactions. 

Generally speaking, there are two competitive processes that occur inside the photocatalyst and affect H_2_ evolution. Similarly as in the case of water purification, first is the charge recombination process. Such a process reduces the excited charges for >90%, as mentioned above [14]; according to some authors, even less than 1% of photoexcited charge carriers are able to participate in the photo-redox reactions forming H_2_ [111]. Such a negative tendency can be improved by controlling the recombination rate [75], as also described in detail in the case of the composite materials used for water purification. The second process is the separation of photogenerated charge carriers that favor H_2_ evolution, also mentioned above in the case of water purification, but here with more important role [111]. 

**Figure 12:**
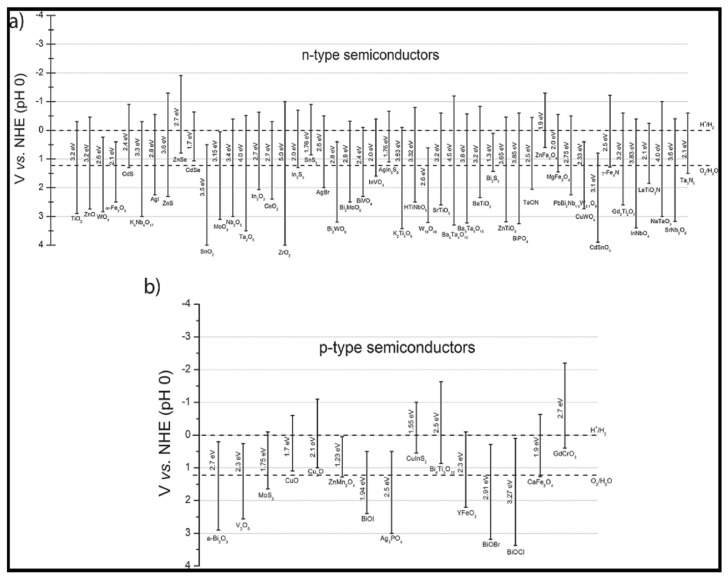
Valence band (VB) and conduction band (CB) band positions of various (**a**) n-type semiconductors; (**b**) p-type semiconductors [111].

The positions of CB and VB define the redox potential of photogenerated charge carriers. A CB minimum (CB_min_) that is smaller than 0 V vs. standard hydrogen electrode (SHE) is required for H_2_ generation, while the maximum of VB (VB_max_) has to be higher than O_2_/H_2_O reduction potential, by definition, in order to enable O_2_ evolution [112]. As mentioned above, H_2_ generation through this process is non-spontaneous, needing the standard Gibbs free energy change of +237 kJ/mol or 1.23 eV, and to accomplish water splitting under visible light irradiation, the bandgap of the photocatalyst should be more than 1.23 eV and less than 3.0 eV [111]. The electronic structures of diverse semiconductors fulfill the necessary conditions for the water splitting reaction, as can be seen from Figure 12. 

**Figure 13:**
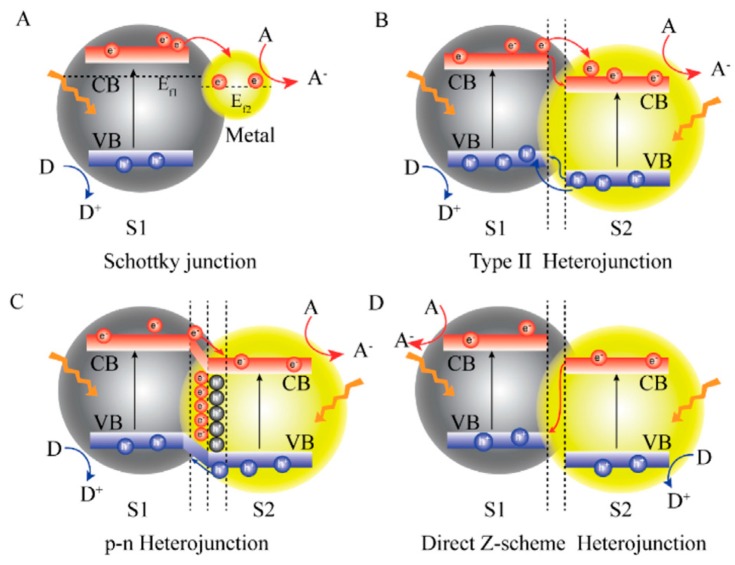
Separation mechanisms of charge carriers in hybrid materials: (**A**) Schottky junction; (**B**) Type II Heterojunction; (**C**) *p-n* Heterojunction; and, (**D**) Direct Z-scheme Heterojunction [112].

Within the scope of this review are recent achievements in TiO_2_-heterojunction systems for photocatalytic H_2_ generation. It is important to explain the separation mechanisms of charge carriers that occur in such hybrid materials: (i) *Schottky junctions*—photogenerated *e*^−^ migration from semiconductor to metal surface due to a higher work function of metal than those of semiconductor, thus forming a Schottky junction (Figure 13a); (ii) *Type II heterojunction* (represented in details in the case of water purification) (Figure 13b); and, (iii) *p-n Heterojunction*—supply of an additional electric field to accelerate the charge carrier transfer (Figure 13c); and, (iv) *Direct Z-scheme heterojunction*—*e*^−^ in the CB of second semiconductor recombined with the photogenerated *h*^+^ in the VB of the first semiconductor, leaving the photogenerated *e*^−^ in first semiconductor and the photogenerated *h*^+^ in second semiconductor for photocatalysis (Figure 13d) [112]. 

The process efficiency is determined through the *Quantum yield* (QY) and *Apparent Quantum Yield* (AQY), as described with Equations (2) and (3) [93]. The overall quantum yield is predicted to be higher than the apparent one since the number of absorbed photons is usually less than that of incident photons [111].

(2) $$\mathrm{QY}\;(\%)=\frac{Number\;of\;reacted\;electrons}{Number\;of\;absorbed\;photons}\times 100=\frac{2\;x\;Nubmer\;of\;hydrogen\;molecules}{Number\;of\;absorbed\;photons}\times 10$$

(3) $$\mathrm{AQY}\;(\%)=\frac{Nubmer\;of\;reacted\;electrons}{Number\;of\;incident\;photons}\times 100=\frac{2\;x\;Numbmer\;of\;hydrogen\;molecules}{Number\;of\;incident\;photons}\times 100$$

H_2_ generation can also be realized in the presence of sacrificial agents, which, in this case, serve as electron donors that accept photogenerated *h*^+^ of the VB, thus enhancing the separation of photogenerated charge carriers, which results in higher quantum efficiency [113]. Alcohols are generally used as a *h*^+^ scavenger, and the more α-H atoms the alcohol has, the higher H_2_ production rate is achieved due to more efficient consumption of *h*^+^ in the photoreaction. The number of α-H atoms in the alcohols can serve as the reference when selecting an appropriate scavenger for photocatalytic reaction [112]. 

After briefly providing the basic principles to fully understand the H_2_ evolution through photocatalytic water splitting, following sections are more focused on the recent achievements in fabrication and the evaluation of different TiO_2_-based heterojunctions with different families of materials, including carbon-based, transition metal oxides and chalcogenides, and multiple-based composites consisting of three or more semiconductor materials for H_2_ generation. 

### 3.1. Carbon-Based/TiO_2_

Among a variety of materials that are selected for the preparation of TiO_2_-based nanocomposites to increase their photocatalytic efficiency, nanostructured carbon materials, such as carbon nanotubes and graphene family nanomaterials (e.g., GO, rGO, g-C_3_N_4_), are of particular interest [114]. The advantages, such as chemical stability, structural diversity with prominent light-absorptive, and electron transport properties, make them promising materials for use in photocatalytic H_2_ generation by the water splitting processes [115]. 

#### 3.1.1. TiO_2_/g-C_3_N_4_

The advantages and limitations of g-C_3_N_4_ are already mentioned above in the case of water treatment. The limitations referring to low light utilization efficiency and insufficient surface area can be easily broken by the preparation of 2D nanomaterials, especially g-C_3_N_4_ nanosheets (CNNS) [84]. The self-assembly method of construction 2D/2D TiO_2_/CNNS heterojunction composites achieved a hydrogen evolution rate (HER) of 350 µmol/h/g under visible light, in comparison with the produced H_2_ with the use of pure TiO_2_ nanosheets (20 µmol/h/g) and g-C_3_N_4_ nanosheets (130 µmol/h/g) [85]. 

Liu et al. recorded another use of CNNS [84], who synthesized partially reduced TiO_2−x_ through NaBH_4_ treatments with the formation of an additional mid-gap band state (Ti^3+^ and oxygen vacancies—O_vs_) to extend absorption edge. The implementation of novel design tactic in the form of a protective carbon layer that was coated onto TiO_2−x_/CNNS hetero-junction photocatalyst enhanced the photocatalytic efficiency. The H_2_ evolution was tested under visible and simulated solar light with the use of triethanolamine (TEOA) as a sacrificial agent and Pt as a co-catalyst. In the case of visible light irradiation, the highest HER was 417.24 µmol/h/g, while, under AM 1.5 irradiation, the obtained amount was 1830.93 µmol/h/g. The enhanced photocatalytic activity that was ascribed to the formation of Ti^3+^ defects was also noticed with the use of g-C_3_N_4_/Ti^3+^-doped TiO_2_ Z-scheme system that was synthesized via the polycondensation of urea with TiO_2_, followed by hydrogenation treatment [86]. UV-Vis diffuse reflectance spectroscopy, X-ray photoelectron spectroscopy (XPS), and electron paramagnetic resonance (EPR) have shown that hydrogenation treatment conferred Ti^3+^ defect states that were below the CB_min_ of TiO_2_ and improved the visible light absorption of the composite with the obtained HER of 1938 µmol/h/g under solar light. 

Although special efforts are being made to synthesize noble-metal free nanocomposites, there is still widespread use of Pt as a co-catalyst in H_2_ evolution reactions. Except for the already mentioned TiO_2−x_/CNNS photocatalyst [84], TiO_2_/g-C_3_N_4_ composites with the use of photodeposited Pt as co-catalyst reached HER of 4128 µmol/h/g [87] and 1041 µmol/h/g [83] under solar and visible light irradiation, respectively. Pan et al. [88] also exhibited a high HER of 13800 µmol/h/m^2^ by the use of Pt as a co-catalyst with g-C_3_N_4_/TiO_2_ nanofilm. Enhanced activity is also attributed to the use of a magnetic-driven rotating frame, which was developed to enhance the mass transfer process during the photocatalytic reaction. 

The charge transfer efficiency between TiO_2_/g-C_3_N_4_ composite can be enhanced by the doping of different heteroatoms, like C and K atoms. Hence, Zou et al. [89] synthesized C-doped TiO_2_@g-C_3_N_4_ core-shell hollow nanospheres with enhanced visible-light photocatalytic activity for H_2_ evolution of 35.6 µmol/h/g, which was 22.7 and 10.5 times higher than that of C-TiO_2_ and g-C_3_N_4_. The structure of TiO_2_ hollow spheres resulted in the reflection of light within the interior cavity, thus increasing the utilization of the light energy. Ma et al. [90] prepared a series of K intercalated g-C_3_N_4_ modified TiO_2_ nanobelts with enhanced light absorption, transfer efficiency, and H_2_ evolution efficiency of 50 µmol/h, which is 6.4 times greater than that of pristine g-C_3_N_4_. The use of carbon atoms in the form of carbon quantum dots (CQDs) as electron reservoirs improves the efficiency of separating the photogenerated charge carriers. CQDs present an important class of carbon materials since their discovery in 2004 by Xu et al. [116], with varying sizes in the range of 1–10 nm. They are good materials for photocatalytic applications due to features, like superiority in chemical stability and low toxicity [117]. Pan et al. synthesized he 2D carbon quantum dots modified porous g-C_3_N_4_/TiO_2_ nano-heterojunction [91] and reached 6.497 µmol/h/g of produced H_2_ with the full spectrum absorption. 

#### 3.1.2. TiO_2_-G/GO/rGO

Following above-mentioned hot-electron mechanism, which can promote redox reactions, Lu et al. explored 3D graphene materials (3DG) coupled with TiO_2_ [76] for efficient photocatalytic H_2_ production under UV-visible light. TiO_2_/3DG with a 5 wt.% graphene loading that was annealed at 650 °C exhibited the highest H_2_ evolution rate of 1205 µmol/h/g. 

**Figure 14:**
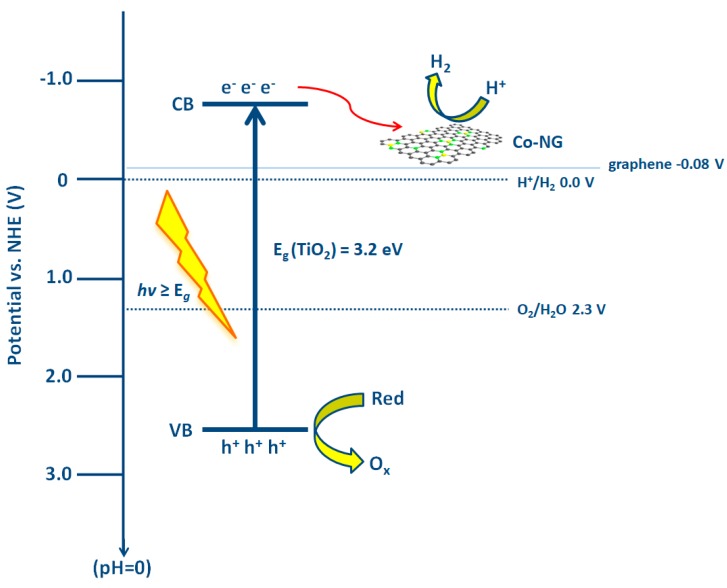
Schematic diagram of proposed photocatalytic mechanism in the CO-NG/TiO_2_ system.

Yi et al. [118] synthesized a composite in which TiO_2_ nanobelts were supported by N-doped graphene (NG) coordinated with a single Co atom to replace noble metals with a cost-effective photocatalyst. Under simulated solar irradiation (Figure 14), *e*^−^/*h*^+^ pairs are formed. The transfer of photogenerated *e*^−^ from the CB of TiO_2_ to Co-NG was energetically favorable since the Fermi energy level of graphene (−0.08V vs. NHE) is lower than the CB of TiO_2_ (−0.39 V vs. NHE). NG, with a large specific surface area, acted as “freeway” for *e*^−^ transportation, delivering *e*^−^ from TiO_2_ to Co single-atom, where they were trapped catalyzing H^+^ reduction to form H_2_ due to lower the overpotential needed for Co-NG when comparing to that of NG. Co-NG/TiO_2_ showed HER of 677.44 µmol/h/g under the illumination of AM 1.5 G simulated sunlight.

GO/TiO_2_ nanocomposites have recently been used for H_2_ production *via* photocatalytic water splitting under visible light through the formation of Ti-O-C bonding by unpaired π electron of GO with TiO_2_ surface [114,119]. GO acts as an *e*^−^ acceptor, promoting the separation of the photogenerated *e*^−^/*h*^+^ pairs in TiO_2_. These nanocomposites can be synthesized by photo assisted reduction via mixing or sonication and by sol-gel [114]. Hernández-Majalca et al. [114] enhanced the synthesis for the GO-TiO_2_ nanocomposite using photoassisted anchoring and modifying GO oxidation method through the use of microwaves. The obtained nonporous product had a specific surface area of 45 m^2^/g and absorption onset of 477 nm, which made it active under visible light. Finally, the photocatalytic activity of the nanocomposite was enhanced towards the production of H_2_, reaching 6500 mol/g in 8 h, which was much higher amount when comparing to that obtained by TiO_2_-P25 (460 mol/g) at the same irradiation time. 

The reduced form of GO, rGO, is a two-dimensional carbon material with the role of an electron mediator that is much superior in chemical stability and morphological diversity than GO [120]. Iwase et al. published the very first report using rGO as a *e*^−^ mediator in 2011 [121]. Since then, a number of published works were recorded for the use of rGO-based composites in photocatalytic H_2_ production [75,122,123]. Recent achievements in the synthesis of TiO_2_/rGO composites for the purpose of H_2_ generation include work from Reedy et al. [75] and Samal et al. [122]. They obtained rather high HERs while using TiO_2_/rGO composites under solar and visible light: 24880 µmol/h/g and 2700 µmol/h/g of produced H_2_, respectively. Ida et al. undertook further investigation on TiO_2_/rGO composites [123], managing to enhance the photocatalytic activity of the obtained composite by the simultaneous doping of nitrogen on TiO_2_ and rGO. The following values for the HER are obtained: TiO_2_ (1585 µmol/h/g) < N-TiO_2_ (6179 µmol/h/g) < TiO_2_/RGO (12244 µmol/h/g) < N-TiO_2_/N-RGO (15028 µmol/h/g).

#### 3.1.3. TiO_2_/CNT 

Recently, TiO_2_/carbon nanotubes (CNT) have been of great interest due to their high-quality active sites, large specific surface area, and retention of charge recombination, where CNTs can act as a *p*-type semiconductor, having a role as a powerful electron sink [119]. The coupling of CNT with TiO_2_ forms an advanced nanocomposite with enhanced quantum efficiency that forms heterojunction acting as an impurity by forming Ti-O-C or Ti-C defects that enable visible light absorption and, consequently, the creation of *e*^−^*/h*^+^ pairs and hindering *e*^−^/*h*^+^ recombination [124]. CNTs, such as single-wall carbon nanotubes (SWCNTs) and multiwall carbon nanotubes (MWCNTs), have attracted much interest due to their unique chemical, electrical, and optical properties [125]. Olowoyo et al. [124] prepared a series of TiO_2_ nanoparticles that were modified with MWCNTs by a combined sonothermal-hydrothermal method. The synthesized photocatalysts were examined for water splitting under batch conditions at different pH ranges. The highest rate of H_2_ yield, amounting 69.41 µmol/h/g, was obtained using 2 wt.% CNT-TiO_2_ under visible light at pH 2. Hence, the acidic medium improved the photocatalytic feasibility of the system due to a higher concentration of H^+^ ions, serving as the reactants, thus increasing the reaction rate.

Bellamkonda et al. [126] used a different approach in synthesizing CNT-G-TiO_2_ composites and prepared nanocomposites via the solution-based method, in which nanocrystalline anatase TiO_2_ was grown onto graphene nanosheets and carbon nanotubes. Spectroscopic and photocatalytic studies revealed that graphene acts as an electron reservoir, while the role of CNTs is to prevent the restacking of graphene nanosheets and provide additional electron transport channels, thereby suppressing the recombination rate of *e*^−^/*h*^+^ pairs in the obtained composite. The combination of all these factors resulted in increasing the HER from 19000 µmol/h/g (obtained by anatase TiO_2_) to 22000 µmol/h/g (obtained by G-TiO_2_), and finally to 29000 µmol/h/g (obtained by CNT-G-TiO_2_), which is 8-fold higher than obtained by the commercial TiO_2_ (Degussa P25). 

The photocatalytic performance of TiO_2_ under visible light can be promoted by coupling both MWCNTs and SWCNTs, as presented by Umer et al. [125]. Such an effect occurs due to their dual natural behavior, such as reducing rapid recombination of *e*^−^/*h*^+^ pairs and providing support in harvesting visible light. The maximum H_2_ evolution rate of 5486 µmol/h/g was achieved over MWCNT/TiO_2_/SWCNT, which is 1.24– and 1.42–fold higher than using single CTN-TiO_2_ composites (SWCNT/TiO_2_ and MWCNT/TiO_2_, respectively).

### 3.2. Transition-Metal Oxides/TiO_2_

Excellent chemical stability has opened the possibility of the application of transition metal oxides (TMO) in the field of clean energy production. The above displayed Figure 12 contains main TMOs, like *p*-type (CuO, V_2_O_5_) and *n*-type (TiO_2_, WO_3_, MoO_3_, ZnO, Fe_2_O_3_) semiconductors that are used in photocatalytic H_2_ production with pertaining VB and CB energy levels. Visible-light driven TMOs with narrow band gaps are highly desired. The most used materials within this group, such as CuO, Fe_2_O_3_, and WO_3_, have the bandgap energies that allow for them to be active in the visible light region, but the low energy levels of CB position disable them from consuming photoinduced electrons in reactions yielding H_2_. By changing the morphologies of desired components and co-doping with different elements, their CB and VB edges can be shifted toward a H_2_ reduction and O_2_ oxidation potential [127]. 

Some TMOs, specifically WO_3_, have been loaded with a different co-catalyst, like Rh, to effectively produce H_2_ from water, to control the desired morphology in the form of nanorods, nanotubes, and nanowires. Camposeco et al. [128] focused on the use of Rh-WO_3_ photocatalyst that was supported on TiO_2_ nanotubes (Rh-WO_3_/NT) for H_2_ production via the water splitting process. WO_3_ alone cannot take part in H_2_ production since the CB energy level of WO_3_ is lower than H_2_ reduction potential. However, by loading with Rh nanoparticles, the enhancement in H_2_ production was noticed. An analysis of energy band levels for the VB and CB that were determined by UV-Vis results and XPS spectra showed that the presence of WO_3_ and Rh in the titanate nanotubes simultaneously shift the VB_max_ and CB_min_, thus reducing the bandgap of titanate nanotubes. 0.5 wt.% Rh– 3 wt.% WO_3_/NT nanocomposite under visible light irradiation yielded HER of 87 μmol/h, while 3 wt.% WO_3_/NT showed much lower effectiveness (only 13 μmol/h). 

In another work, Ren et al. [129] constructed cooperative Schottky and *p-n* (SPN) heterojunction by forming a NiO/Ni/TiO_2_ heterostructure that showed a narrower band gap, higher photocurrent density, and ability to absorb light in the visible region. High HER is obtained for the observed composite, amounting 4653 μmol/h/g, which is approx. 2.3 times higher than obtained with NiO/TiO_2_ with a *p-n* junction (2059 μmol/h/g), under visible light irradiation and by the use of Pt as a co-catalyst. 

A representative example of TMOs is also hematite (Fe_2_O_3_), already referred above to as promising visible light active photocatalyst for water treatment. The coupling of Fe_2_O_3_ with TiO_2_ efficiently inhibits the recombination of photogenerated charge carriers and enhances the absorption of solar light [130]. By the use of thermal decomposition of FeCl_3_ and TiCl_4_ as precursors, Bhagya et al. [113] synthesized the Fe_2_O_3_-TiO_2_ composite and investigated its photocatalytic activity for H_2_ production under the influence of different proton sources. Besides focusing on the use of TMOs for improving photocatalytic efficiency, this work also highlights the influence of different sacrificial agents as electron donors that consume photogenerated *h*^+^, yielding H_2_ production. Under simulated solar irradiation, a very high H_2_ rate of 880 μmol/h, with an apparent quantum efficiency of 19.39%, is achieved while using Fe_2_O_3_-TiO_2_ and diethylamine hydrogen chloride (DAH), which is much higher than that obtained in the case without DAH (323 μmol/h). Madhumitha et al. [130] also explored the influence of different sacrificial reagents on H_2_ production under a visible light source. With the optimization of parameters, i.e., catalyst dosage, flow rate, incident light irradiation, and type of sacrificial agent, they achieved the maximum of HER, amounting 2700 μmol/h. The increase in photoactivity was attributed to the effective charge transfer from TiO_2_ to Fe_2_O_3_ and the use of EDTA, which suppressed the recombination of photogenerated charge carriers. 

Easy preparation, environmental friendliness, and good re-utilization enable the wide use of ZnO/TiO_2_-based composites. Xie et al. [131] achieved high rates of H_2_ evolution while using ZnO/TiO_2_ composites with Pt as a co-catalyst. Under visible light irradiation, 2150 µmol/h/g of H_2_ is achieved. Additionally, high carrier mobility can be achieved by the use of ZnO in the form of quantum dots (QDs), which presents ideal heavy-metal free “green” modification of TiO_2_ composites. Chen et al. obtained the fabrication of ZnO QDs decorated TiO_2_ nanowires via a facile calcination method [132]. They used the obtained composite under solar irradiation next to Pt as a co-catalyst and achieved HER of 313.5 µmol/h. 

The use of TMO QDs is also recorded in the work of Liu et al. [133], who decorated two-dimensional TiO_2_ nanobelts with zero-dimensional Co_3_O_4_ quantum dots. When compared with bulk materials, 0D Co_3_O_4_ QDs have attracted considerable attention due to their small size (<10 nm), providing a large specific surface area with more active sites and shorter charges transport paths. Due to the decoration of Co_3_O_4_ QDs, the bandgap of the obtained composite was also narrowed, and in application next to Pt as co-catalyst, they obtained a rather high HER of 1735.1 μmol/h/g. In comparison, Zhang et al. [134], with the use of bulk p-Co_3_O_4_/n-TiO_2_, achieved a smaller HER of 8.16 μmol/h/g.

Among the TMOs that appear as promising candidates for coupling with TiO_2_, CuO is one of such, especially due to its narrow bandgap (1.4–1.6 eV) and the promotion of effective charge separation [5,135], as already reported as promising water treatment photocatalyst. Hasan et al. [5] conducted solar H_2_ production using a TiO_2_/CuO nanofiber composite that was synthesized by electrospinning technique. Fabricated nanofibers were annealed in different atmospheres to determine the crystalline phase and photocatalytic performance. For the nanofibers that were crystallized in the anatase phase, EPR and XRD analysis referred to the substitution of some Ti^4+^ ions by Cu^2+^ ions, leading to the formation of some defects below the CB of TiO_2_, which led to a narrow band gap, yielding enhanced HER in the amount of 2715 µmol/h/g. For comparison, without annealing in a different atmosphere, Wang et al. [135] only achieved 47 µmol/h/g of produced H_2_ while using the TiO_2_/CuO composite that was irradiated by solar light. Other oxidation states of Cu inside the oxides are investigated for coupling with TiO_2_, such as Cu_2_O [136]. With all of the benefits, such as environmental compatibility, high visible light activity and earth abundance, wider applications of Cu_2_O in water splitting are still limiting, since the redox potential of monovalent copper lies within its band gap, thus photogenerated charge carriers thermodynamically prefer the transformation of Cu_2_O into CuO and Cu, rather than to be used in redox reactions with water constituents forming H_2_ [136]. Wei et al. [136] stabilized the Cu_2_O by modulating the defects in faceted Cu_2_O/TiO_2_ heterostructures to suppress this disproportionation process. Hence, Cu_2_O was arranged onto 101-faceted TiO_2_ and it was found that oxygen vacancies in {101}-faceted TiO_2_ can create a unique channel for Z-scheme charge transfer in Cu_2_O/TiO_2_ heterostructures. Composite that was obtained by such an approach showed the maximum HER of 32600 µmol/h/g, with a quantum efficiency of 53.5% at an irradiation wavelength of 350 nm. 

### 3.3. Transition Metal Chalcogenides-TiO_2_

As already mentioned, various techniques have been applied to modify the TiO_2_ photocatalysts with a purpose of wider application in the field of solar-driven H_2_ production through water splitting. Sensitization with narrow bandgap semiconductors was found to be an effective method for enhancing all of the deficiencies that occur during the sole use of TiO_2_. Regarding an appropriate band gaps, another group of semiconductors with great application potential in photocatalytic H_2_ generation are transition metal chalcogenides. Recently, CdS is one of the most studied materials [54,55,137,138,139,140]; however, the toxic effects due to potential leaching of Cd^2+^ have to be strongly considered, followed by others (MoS_2_, ZnS, ZnSe, CdSe) indicated in Figure 12, as mentioned in part related to water treatment [127]. 

#### 3.3.1. TiO_2_/CdS

CdS is the most important chalcogenides semiconductor as a hydrogen production catalyst due to its narrow bandgap (2.4 eV), which enables its visible light response [56]. Its drawbacks described above in Section 2.2. can be alleviated; susceptibility to photocorrosion can be suppressed by the use of sacrificial agents (sodium sulfite/sulfide) that effectively consume photogenerated *h*^+^, while the limited separation efficiency of photogenerated charge carriers can be solved either by using CdS in the form of QDs due to a shorter transportation path or by incorporating CdS onto support materials, such as TiO_2_ [55,56].

Rao et al. [137] synthesized CdS/TiO_2_ core/shell nanorods with tunable shell thickness to minimize charge carriers recombination and limit photocorrosion. The investigation of photocatalytic activity performed under UV-vis light irradiation confirmed that optimized concentration of sacrificial agents (0.3 M Na_2_S and Na_2_SO_4_ aqueous solution), shell thickness of 6.3 nm, and solution pH of 8.0 enhance the H_2_ production rate of 5791 mL/h/g. Du et al. [138], who fabricated pyramid-like CdS nanoparticles that were grown on porous TiO_2_, obtained the same type of composite, but with the different morphology. Under UV-vis irradiation and without noble-metal co-catalysts, 5 mol% CdS-TiO_2_ achieved an H_2_ production rate of 1048.7 μmol/h/g, which is almost six times and 1.5 times higher than that of pure TiO_2_ and CdS, respectively. Table 10 provides further examples of TiO_2_/CdS-based composites with their respective photocatalytic activities for H_2_ production.

#### 3.3.2. TiO_2_/CuS

CuS has emerged as an alternative co-catalyst for H_2_ production, which is abundant, cheap, and nonhazardous. With its VB and CB at positions of −1.56 eV vs. NHE, and −0.09 eV, CuS shows low reflectance in the visible and relatively high reflectance in the near-infrared region, which makes it a good candidate for solar energy absorption [144]. Chandra et al. [144] investigated the photocatalytic activity for H_2_ generation of synthesized CuS/TiO_2_ (CT) heterostructured nanocomposite under UV-vis and only visible light. Under irradiation, the highest HER of 12362 μmol/h/g was achieved while using 0.4 mol.% CuS/TiO_2_, while only 155 μmol/h/g of H_2_ was produced under visible light and comparable conditions.

In comparison, Dang et al. [145] produced a series of Cu_x_S (x = 1 or 2) co-modified TiO_2_ nanocomposites while using a one-step precipitation approach. The EDS, XPS, and XRF results confirmed the existence of three different phases: CuS, Cu_2_S, and TiO_2_. The results have shown that CuS and Cu_2_S dual co-catalysts under simulated solar light exhibited high H_2_ production of 5620 µmol/h/g, which is about 58 times higher than that of the unloaded TiO_2_. The enhancement in H_2_ production can be contributed to the co-deposition of CuS and Cu_2_S onto the TiO_2_ nanoparticle surface that efficiently extended visible light absorption and facilitated the separation of charge carriers. The Cu_x_S/TiO_2_ composite showed high stability; after three consecutive cycles the photocatalytic efficiency for H_2_ production decreased for only 11.7%. 

#### 3.3.3. TiO_2_/MoS_2_

**Figure 15:**
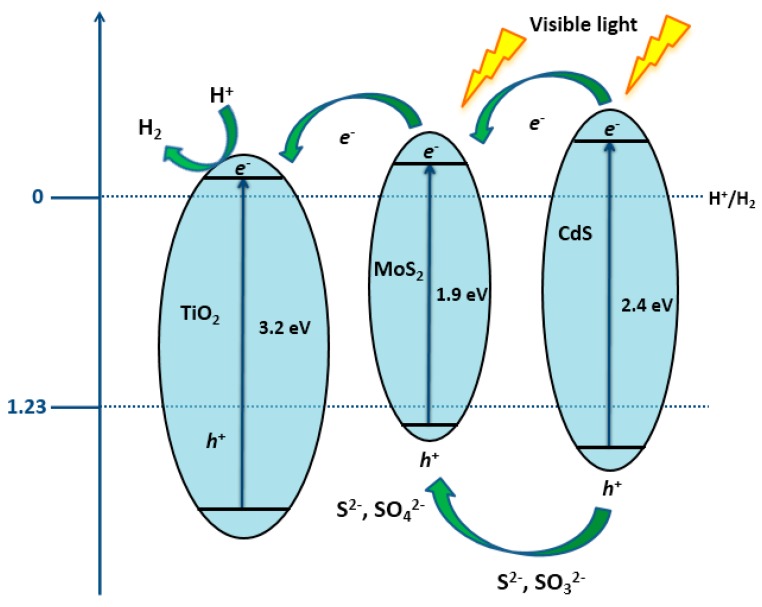
The proposed photocatalytic mechanism in MoS_2_-CdS-TiO_2_ photocatalyst.

MoS_2_ represents two-dimensional transition metal dichalcogenide (TMD) that can be prepared into ultrathin-layered structures and, thus, participates in the H_2_ evolution reaction as an effective non-noble metal alternative [117,146]. Without the use of any sacrificial agent and co-catalyst, Huang et al. [146] observed photocatalytic H_2_ generation while using MoS_2_ quantum dots@TiO_2_ nanotube arrays nanocomposite in pure water under visible light. The photocatalytic activity was influenced by the amount of MoS_2_ QDs coated on TiO_2_ NTAs. Hence, the maximum of 53.9 μmol/cm^2^/h of H_2_ was produced, which was ascribed to the decreased bandgap and the surface plasmonic properties of the obtained composite promoting charge carrier separation and the absorption capacity to visible light. Du et al. [63] grew *in situ* two transition metal chalcogenides—MoS_2_ and CdS—on porous TiO_2_ by using the sol-gel method, followed by the calcination and hydrothermal method. Under visible light irradiation and without the use of noble metals as the co-catalyst, 3% MoS_2_-CdS-TiO_2_ produced 4146 μmol/h/g of H_2_. In this ternary composite (Figure 15), the porous structure of TiO_2_ accepts generated *e*^−^ from CdS and provides surface area for H_2_ production, while MoS_2_, as a conductive medium, enabled the transfer of *e*^−^ between CB of CdS and TiO_2_, simultaneously inhibiting the photocorrosion of CdS as *h*^+^ collector.

### 3.4. Multiple TiO_2_-Based Composites 

This section is dedicated to multiple ternary and quaternary TiO_2_-based composites. Recently, significant emphasis was laid on the formation of Z-scheme structured photocatalysts. In such materials, redox reactions occur in each semiconductor, allowing for the combination of a semiconductor with strong reduction power with another semiconductor with strong oxidation power [93].

All of the solid Z-scheme heterojunctions are usually composed of two photocatalysts and an electron mediator, which enable efficient H_2_ production through the synergistic action between two isolated photosystems and electron mediator cleverly arranged in a nano-platform [147]. Reversible redox couples (e.g., IO_3_^−^/I^−^, Fe^3+^/Fe^2+^) are usually applied as electron mediators in the Z-scheme system. Solid electron mediators are more suitable for the application. Noble metals (Au, Ag) and carbon-based materials (MWCNT, rGO, CQDs) are commonly used as solid electron mediators for photocatalytic H_2_ generation [148]. 

**Figure 16:**
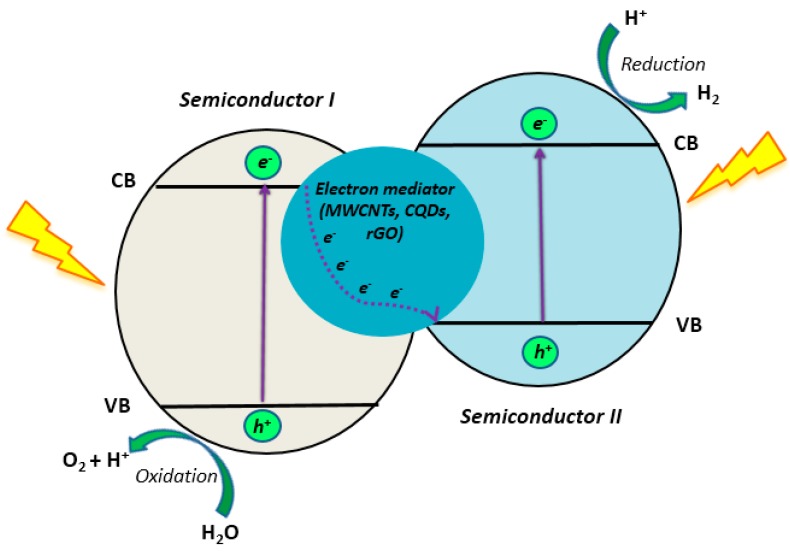
The type Z-heterojunction with the use of different solid electron mediators (multiwall carbon nanotubes (MWCNTs), carbon quantum dots (CQDs), rGO).

Ng et al. [147], who synthesized Zn_0.5_Cd_0.5_S-MWCNT-TiO_2_ ternary nanocomposite, where MWCNT acted as an electron mediator, fabricated a solid Z-scheme system. The obtained material efficiently suppressed charge recombination and promoted water reduction, achieving HER of 21.9 µmol/h. Furthermore, Liu et al. [149] have used carbon quantum dots (QDs) as an electron mediator between TiO_2_ and Zn_0.5_Cd_0.5_S film and achieved 38740 µmol/h/m^2^ of produced H_2_ under solar light. Lv et al. investigated the use of rGO as an electron mediator [120], synthesizing sandwich-like TiO_2_/rGO/LaFeO_3_ ternary heterostructure, which, under solar light, obtained HER of 893 µmol/h/g, which is almost 3.2, 14.4, and 11.4 times superior to the direct Z-scheme components TiO_2_/LaFeO_3_ composite, pure TiO_2_, and LaFeO_3_, respectively. The photocatalytic mechanism of H_2_ production is the same for all three above mentioned solid electron mediators: MWCNT, rGO, and CQDs (Figure 16). Upon excitation by light, *e*^−^/*h*^+^ are formed in Z-scheme semiconducting components, depending on the ability of each component to absorb emitted light. Hence, photogenerated *e*^−^ from semiconductor I can be easily recombined with *h*^+^ from semiconductor II through the electron mediator, leaving more oxidative holes and reductive electrons to participate in the redox reactions in the corresponding active sites [148].

In Z-type heterojunction, noble metals can also perform the role of electron mediators. Zou et al. performed the construction of g-C**_3_**N**_4_**/Au/C-TiO**_2_** hollow spheres with Au nanoparticles (NPs) as the electron mediator [150]. The as-prepared composite showed high HER of 129 µmol/h/g under visible light, which can be attributed to the efficient charge separation in the constructed Z-scheme system, the broadened visible-light response range, owing to the surface plasmon resonance (SPR) effects on Au nanoparticles, and the hollow structure of C-TiO_2_ that gives photocatalyst unique properties of low density and high light-harvesting efficiency. In another work presented by Yang et al. [151], Au NPs were applied as a solid electron mediator in the ternary urchin-like ZnIn_2_S_4_-Au-TiO_2_ nanocomposite, which, at an optimal ratio of 24 wt.% Au NPs and 60 wt.% ZnIn_2_S_4_, achieved HER of 186.3 µmol/h/g under solar light irradiation.

Table 11 lists other multiple composites, except ones with the Z-scheme heterojunction that are already mentioned, as well as reaction conditions in the photocatalytic system and the obtained hydrogen evolution rates. 

## 4. Conclusions

TiO_2_-based photocatalytic technology represents a promising up-coming technology for both renewable energy generation and water purification applications. It is necessary to work on developing TiO_2_-based semiconductor materials, which are active under broader spectrum of solar light, overcoming the indicated disadvantages of the solely TiO_2_ utilization, since TiO_2_ as a photocatalytic material cannot be used alone due to its limitations such as activity only in UV light region and rapid recombination of photogenerated charge carriers. 

TiO_2_-semiconductor coupling offers promising results in water purification, particularly for the degradation and mineralization of CECs. However, it is necessary to evaluate the toxicities of degradation intermediates of CEC to check the real efficiency of such composites. Furthermore, the immobilization of TiO_2_–Semiconductor composites to photocatalytic reaction membranes must be envisaged for further upscale opportunities.

Great progress has also been recorded in the development of TiO_2_-based heterojunction for the application in solar-driven photocatalytic hydrogen generation. By morphological control of the obtained composites, higher H_2_ generation, as well as better light harvesting can be achieved. It is noticed that still a great deal of research is being conducted by the use of different noble-metal co-catalysts and sacrificial agents, which, although increasing the efficiency of the process, reduces its environmental friendliness and increases the performance cost. Accordingly, to allow for the practical deployment of such units, as well as commercialization, it is necessary to produce cost-effective systems that will consider economic impact assessment, including operation cost and energy consumption, and that will not require the use of costly co-catalysts and other substances that promote system performance. 

## Figures and Tables

**Table 1:** Photocatalytic degradation of contaminants of emerging concern (CECs) over TiO_2_/WO_3_ composites.

Catalyst	Target Pollutant	Initial Concentration/Working Volume ((mg L^−1^) /mL)	Experimental Conditions	Reaction Time	Removal Extent (%)	Reference
TiO_2_ - WO_3_ (0.5 g/L )	2,4-dichlorophenoxy acetic acid	50 (in 250 mL)	Light Source: natural sunlight 11AM-4PM; pH = 4	120 min	94.6 (TOC = 88.6)	[24]
TiO_2_ - WO_3_ (0.6 g/L)	Diclofenac	25 (in 100 mL)	Light Source: 400 W Metal Halide Lamp; pH = 5	240 min	TOC = 91	[30]
TiO_2_ - WO_3_ (0.1 g/L)	Amoxicillin	100 (in 25,000 mL)	CPC Reactor with accumulated energy 550,000 J/m^2^	NA	64.4 (@ 550 kJ/m^2^)	[35]
(WO_3_/TiO_2_-C) (1.0 g/L)	Diclofenac	10 (in 300 mL)	Light Source: 1500 W Xenon Lamp with filter(λ > 290 nm) ; pH = 7	NA	100 (@ 250 kJ/m^2^) (TOC = 82.4 @ 400 kJ/m^2^)	[32]
(WO_3_/TiO_2_-N) (1.0 g/L)	Diclofenac	10	Light Source: 1500 W Xenon Lamp with ID65 solar filter; pH = 6.5	NA	100 (@ 250 kJ/m^2^) (TOC = 100 @400 kJ/m^2^)	[31]

NA—not available.

**Table 2:** Photocatalytic degradation of CEC’s over TiO_2_/Fe_2_O_3_ composites.

Catalyst	Target Pollutant	Initial Concentration/Working Volume ((mg L^−1^) /mL)	Experimental Conditions	Reaction Time	Removal Extent (%)	Reference
TiO_2_/Fe_2_O_3_ (0.1 g/L )	Diazinon	10 (in 300 mL)	Light Source: 60 W Philips Visible lamp; pH = natural	45 min	95.07	[37]
TiO_2_/Fe_2_O_3_ (10 mg)	2,4-dichlorophenoxy acetic acid	50 (in 100 mL)	Light Source: 300 W Xenon Lamp; pH = natural	120 min	100 (TOC = 100 @ 150 min.)	[23]
TiO_2_/Fe_2_O_3_ (0.5 g/L )	2,4-dichlorophenoxy acetic acid	50 (in 250 mL)	Light Source: natural sunlight 11AM-4PM; pH = 4	120 min	96.8 (TOC =90 @ 240 min.)	[24]
TiO_2_/Fe_2_O_3_ (70 mg)	Oxytetracycline Hydrochloride	60 (in 70 mL)	Light Source: 300 W Iodine Tungsten Lamp; pH = 5.5	300 min	75.6	[38]
TiO_2_/Fe_2_O_3_ (1.0 g/L)	Oxytetracycline	60	Light Source: 300 W Iodine Tungsten Lamp; pH = 5.5	300 min	~80	[39]
TiO_2_/Fe_2_O_3_/CNT (100 mg)	Tetracycline	20 (in 100mL)	Light Source: 300 W Xenon Lamp; pH = natural	90 min	89.41	[40]
TiO_2_-coated α-Fe_2_O_3_ core-shell (100 mg)	Tetracycline Hydrochloride	50 (in 200 mL)	Light Source: 300 W Xenon Lamp (λ > 420 nm) ; pH = 5. 45 Oxidant = 120 µL (30% H_2_O_2_)	90 min	100	[41]

**Table 3:** Photocatalytic degradation of CEC’s over TiO_2_/MFe_2_O_4_ composites.

Catalyst	Target Pollutant	Initial Concentration/ Working Volume ((mg L^−1^) /mL)	Experimental Conditions	Reaction Time	Removal Extent (%)	reference
N-TiO_2_/ CaFe_2_O_4_ /diatomite (2.0 g/L)	Tetracycline	10 (in 200 mL)	Light Source: 150 W Xenon Lamp with UV light filter	150 min	91.7 (TOC =~80 @ 2h)	[44]
N-TiO_2_/ SrFe_2_O_4_ /diatomite (2.0 g/L)	Tetracycline	10 (in 200 mL)	Light Source: 150 W Xenon Lamp with UV light filter	150 min	92 (TOC = ~80 @ 2h)	[46]
Ce/N co-doped TiO_2_ / NiFe_2_O_4_ diatomite (0.5 g/L)	Tetracycline	20 (in 200 mL)	Light Source: 150 W Xenon Lamp with UV light filter	180 min	98.2 (TOC = ~95)	[45]
ZnFe_2_O_4_ / TiO_2_ (1.0 g/L)	Bisphenol A	10 (in 200 mL)	Light Source: 300 W Xenon Lamp pH= 7	30 min	100	[47]

**Table 4:** Photocatalytic degradation of CEC’s over TiO_2_/Cu_2_O composites.

Catalyst	Target Pollutant	Initial Concentration/ Working Volume ((mg L^−1^) /mL)	Experimental Conditions	Reaction Time	Removal Extent (%)	Reference
Cu_2_O-TiO_2_ supported palygorskite (1.0 g/L)	Tetracycline Hydrochloride	30 (in 50mL)	Light Source: 500 Xe Lamp; pH = 8.7	240 min	88.81	[48]
TiO_2_-Cu_2_O film	Tetrabromodiphenyl Ethers	5 (in 100 mL)	Light Source: 300 W Xenon Lamp; pH = natural solvent CH_3_OH:H_2_O (50:50 v/v)	150 min	90	[49]

**Table 5:** Photocatalytic degradation of CEC’s over TiO_2_/Bi_2_O_3_ composites.

Catalyst	Target Pollutant	Initial Concentration/ Working Volume ((mg L^−1^) /mL)	Experimental Conditions	Reaction Time	Removal Extent (%)	Reference
Bi_2_O_3_–TiO_2_ (50 mg)	Quinalphos	25 (in 50 mL)	Light Source: Visible light with 1.56µmol/m^2^/s; pH = 8	100 min	92	[50]
Bi_2_O_3_–TiO_2_ (0.5 g/L)	Ofloxacin	25	Light Source: 70.3 K lux; pH = 7	120 min	92	[51]

**Table 6:** Photocatalytic degradation of CEC’s over TiO_2_ /Metal sulfide composites.

Catalyst	Target Pollutant	Initial Concentration/ Working Volume ((mg L^−1^)/mL)	Experimental Conditions	Reaction Time	Removal Extent (%)	Reference
CdS –TiO_2_ (50 mg)	Tetracycline Hydrochloride	50 (in 50mL)	Light Source: 500 W Xenon Lamp with filter (λ > 400 nm); pH = natural	480 min	87.0	[58]
Au-CdS/TiO_2_ nanowire (20 mg)	Ciprofloxacin	20	Average solar light intensity = 100, 000	60 min	99	[57]
CdS/TiO_2_ (450 mg)	Ofloxacin	10 (in 100mL)	Light Source: 85 W Oreva bulb with 4150 lumens (λ = 450-650 nm); pH = natural	180 min	86	[52]
CdS nano-rod/TiO_2_ nano-belt ( 0.50 g/L)	17α-ethynylestradiol	3 (in 10 mL)	Light Source: 500 W Xenon Lamp with filter (λ > 420 nm); pH = natural	120 min	92	[25]
CuS/TiO_2_ nanobelts	Enrofloxacin	5 (in 35 mL)	Light Source: 35 W Xenon Lamp; pH = natural	120 min	85.5 (TOC = 27.7)	[59]
Au-CuS-TiO_2_ nanobelts	Oxytetracycline	5 ( in 35 mL)	Light Source: 35 W Xenon Lamp; pH = natural	60 min	96 (TOC = 68)	[60]
MoS_2_ /TiO_2_ (25 mg/L)	Acetaminophen	302	Light Source: Sunlight; pH = natural	25 min	40	[64]
N,S co-doped TiO_2_ @MoS_2_ (0.98g/L)	Diclofenac	0.15 ( in 100 mL)	Light Source: 60 W LED lamp; pH = 5.5	150 min	98	[61]
TiO_2_/SnS_2_ films	17β-estradiol	1.36 (in 90 mL)	Light Source: 450 W Xenon Arc Lamp	90 min	51.0	[66]
TiO_2_/SnS_2_ films	Diclofenac	31.8 ( in 90mL)	Light Source: 450 W Xenon Arc Lamp; pH = 4	60 min	76.21	[67]

**Table 7:** Photocatalytic degradation of CEC’s over TiO_2_/Silver-Based Semiconductor composites.

Catalyst	Target Pollutant	Initial Concentration/ Working Volume ((mg L^−1^)/mL)	Experimental Conditions	Reaction Time	Removal Extent (%)	Reference
Ti^3+^ -doped TiO_2_ nanotubes/ Ag_3_PO_4_ quantum dots (0.5 g/L)	Tetracycline	10 (NA)	Light Source: 400 W Xenon Lamp; pH = natural	8 min	90	[72]
TiO_2_ nanotube/ Ag_3_PO_4_ nanoparticles (40 mg)	Ciprofloxacin	10 (in 40 mL)	Light Source: 300 W Xenon Lamp	60 min	85.3	[73]
TiO_2_-x / Ag_3_PO_4_ (100 mg)	Bisphenol A	10 (in 100 mL)	Light Source: 500 W Xenon Lamp with filter (λ = 420 nm); pH = natural	16 min	95	[74]
Ag_2_O/ TiO_2_ quantum dots (0.25 g/L)	Levofloxacin	10 (in 100 mL)	Light Source: 85 W Oreva CFL (4150 lumens) (λ = 380–700 nm) pH=4	90 min	81	[27]
Ag_2_O /TiO_2_ –zeolite (50 mg)	Norfloxacin	5 (in 100 mL)	Light Source: 35 W Xenon Lamp	60 min	98.7 (TOC = 83.1)	[28]

**Table 8:** Photocatalytic degradation of CEC’s over TiO_2_/Semiconductor/graphene composites.

Catalyst	Target Pollutant	Initial Concentration/ Working Volume ((mg L^−1^)/mL)	Experimental Conditions	Reaction Time	Removal Extent (%)	Reference
TiO_2_/ WO_3_/GO (2 mg)	Bisphenol A	20 ( in 50 mL)	Light Source: sunlight Ph = 7	7 h	93.2	[77]
Graphene-WO_3_ /TiO_2_ nanotube (photoelectrodes )	Dimethyl Phthalate	10 (in 40 mL)	Light Source: 150W Xe lamps	120 min	75.9	[78]
TiO_2_ /ZnO/GO (0.5 g/L)	Bisphenol A	10 (in 50 mL)	Light Source: 18 UV lamps (λ =365 nm) ;Visible metal halide lamps(λ = 400–800 nm) pH = 6	120 min. (UV) 180 min. (Vis)	99.7 (UV) 94.9 (Vis)	[79]
TiO_2_ /ZnO/GO (0.5 g/L)	Ibuprofen	10 (in 50 mL)	Light Source: 18 UV lamps (λ = 365 nm) ;Visible metal halide lamps(λ = 400–800 nm) pH = 6	120 min. (UV) 180 min. (Vis)	98.5 (UV) 79.6 (Vis)	[79]
TiO_2_ /ZnO/GO (0.5 g/L)	Flurbiprofen	10 ( in 50 mL)	Light Source: 18 UV lamps (λ=365 nm) ;Visible metal halide lamps(λ= 400–800 nm) pH= 6	120 min. (UV) 180 min. (Vis)	98.1(UV) 82.2 (Vis)	[79]
ZnFe_2_O_4_/rGO/TiO_2_ (0.1 g)	Fulvic Acid	20 (in 50 mL)	Light Source: 300 W (λ=420 nm); Vol _H_2_O_2__ = 0.8 mL, pH= 7	180 min	95.4%	[80]
TiO_2_ /MoS_2_ /rGO (0.5 g/L)	Bisphenol A	10 (in 50 mL)	Light Source: 20 W (λ = 254 nm);	300 min	62.4	[81]
TiO_2_/BiVO_4_/rGO	Tetracycline	10 µg/L (NA)	Light Source: 1000 W Xe Lamp (λ = 420 nm) with filter	120 min	96.2	[82]
TiO_2_/BiVO_4_/rGO	Chlorotetracycline	10 µg/L (NA)	Light Source: 1000 W Xe Lamp (λ = 420 nm) with filter	120 min	97.5	[82]
TiO_2_/BiVO_4_/rGO	Oxytetracycline	10 µg/L (NA)	Light Source: 1000 W Xe Lamp (λ = 420 nm) with filter	120 min	98.7	[82]
TiO_2_/BiVO_4_/rGO	Doxycycline	10 µg/L (NA)	Light Source: 1000 W Xe Lamp (λ = 420 nm) with filter	120 min	99.6	[82]

**Table 9:** Photocatalytic degradation of CEC’s over TiO_2_ /g-C_3_N_4_ composites.

Catalyst	Target Pollutant	Initial Concentration/ Working Volume ((mg L^−1^)/mL)	Experimental Conditions	Reaction Time	Removal Extent (%)	Reference
g-C_3_N_4_/TiO_2_ (30 mg)	Ciprofloxacin	10 (in 80 mL)	Light Source: 300 W Xe Lamp with filter (λ > 400 nm) pH = natural	180 min	88.1	[96]
g-C_3_N_4_/TiO_2_ (30 mg)	Acyclovir	10 (in 100 mL)	Light Source: 300 W Xe Lamp with filter (λ > 420 nm) pH = natural	90 min	100	[97]
mpg-C_3_N_4_/TiO_2_ (membrane)	Sulfamethoxazole	10 (in 50 mL)	Light Source: 300 W Xe Lamp pH = natural Flow rate = 13 mL/min. Membrane flux = 918 L /m^2^ h	1800 min	69	[98]
TiO_2_@g-C_3_N_4_ core-shell (100 mg)	Tetracycline	20 (in 100 mL)	Light Source: Xenon Lamp with full spectrum pH = natural	9 min	(2.2 mg/min.)	[99]
g-C_3_N_4_ –shielding polyester/ TiO_2_ (130 mg)	sulfaquinoxaline	2 × 10^−5^ mol/L (30 mL)	Light Source: Q-Sun Xe-1 test, pH = 7	90 min	97	[101]
g-C_3_N_4_ –shielding polyester/ TiO_2_ (130 mg)	thiamethoxam	2 × 10^−5^ mol/L (30 mL)	Light Source: Q-Sun Xe-1 test, pH = 7	180 min	~95	[101]
g-C_3_N_4_/TiO_2_/kaolinite (200 mg)	Ciprofloxacin	10 (in 100 mL)	Light Source: Ave. light intensity =90 mW/cm^2^ ; Xe Lamp with filter (λ > 400 nm), pH = natural	240 min	92	[100]
S-Ag/ TiO_2_ @ g-C_3_N_4_ (0.20 g/L)	Triclosan	10 (in 100 mL)	Light Source: 250 W Xe Lamp with filter (λ > 420 nm), pH = 7.8	60 min	92.3 (Detoxification Efficiency= 64.3± 3.9)	[102]
Co-TiO_2_ @g-C_3_N_4_ (5 mg ; 2 × 2 cm^2^ membranes)	Tetracycline Hydrochloride	20 (in 10 mL)	Light Source: 300 W Xe Lamp with filter (λ > 420 nm), pH = 7	60 min.	90.8	[103]
D35-TiO_2_/g-C_3_N_4_ (0.5g/L)	Bisphenol A	10 (in 100 mL)	Light Source: 300 W Metal Halide pH = 7, Oxidant = 2mM Persulfate	15 min	100 (TOC= 50)	[104]
C dots decorated g-C_3_N_4_/ TiO_2_ (1.0 g/L)	Enrofloxacin	4 ( in 50 mL)	Light Source: 350 W Xe Lamp with filter (λ > 420 nm) pH = natural	60 min	91.6	[105]
graphene quantum dots/ Mn-N-TiO_2_ /g-C_3_N_4_ (45 mg)	Ciprofloxacin	10 (in 80 mL)	Light Source: 300 W Xe Lamp (320 ≤λ ≤ 780 nm), pH = 7	120 min	89	[107]
graphene quantum dots/ Mn-N-TiO_2_ /g-C_3_N_4_ (45 mg)	Diethyl Phthalate	10 (in 80mL)	Light Source: 300 W Xe Lamp (320 ≤ λ ≤ 780 nm), pH = 7	120 min	70.4	[107]
MoS_2_ supported TiO_2_/g-C_3_N_4_ (30 mg)	Atrazine	10 (in 100 mL)	Light Source: 500 W Xe Lamp (λ > 420 nm), pH = 7	300 min	86.5	[108]
WO_3_–TiO_2_ @g-C_3_N_4_	Acetylsalicylate	10 (in 100 mL)	Light Source: 500 W Metal Halide pH = natural	90 min	98	[109]
WO_3_–TiO_2_ @g-C_3_N_4_	Methyl-theobromine	10 (in 100 mL)	Light Source: 500 W Metal Halide pH = natural	90 min	97	[109]

**Table 10:** The photocatalytic performance of H_2_ generation in some related TiO_2_/CdS-based nanocomposites.

Photocatalyst	Light Source	HER	Reference
CdS/Pt/TiO_2_ film	300 W Xe lamp	3.074 µmol/h/g	[54]
CdS/Pt/TiO_2_ nanosheets	350 W Xe arc lamp	265 µmol/h	[141]
CdS/Pt/TiO_2_ nanotubes	300 W Xe lamp	402 µmol/h	[142]
CdS/TiO_2_ nanotubes	350 W Xe lamp	2585 µL/h/g	[143]
CdS-Ti-MCM-48-21-25	300 W Xe lamp	2726 µmol/h	[139]

**Table 11:** Multiple TiO_2_-based composites for the use in photocatalytic hydrogen generation.

Photocatalyst	Reaction Conditions	HER	Reference
CdSQDs/WC/TiO_2_	Photocatalyst dispersed in 20 vol.% lactic acid as an electron donor under visible light.	624.9 µmol/h/	[55]
ZnO/ZnCr_2_O_4_@TiO_2_-NTA	Photocatalyst dispersed in aqueous methanol solution under simulated solar light.	1680 µmol/cm^2^	[152]
F-TiO_2_/CdSe-DETA	Photocatalyst was dispersed in a mixed solution of Na_2_S and Na_2_SO_3_ as a sacrificial agents under visible light with the use of Pt as a cocatalyst.	12381 µmol/h/g	[153]
g-C_3_N_4_/TiO_2_/RGO	5 mg of the g-C_3_N_4_-TiO_2_/RGO nano-composite was dispersed in 50 mL glycerol-water solution under UV-vis light.	19610 µmol/h/g	[13]
CDS/CNF/Pt-TiO_2_	Photocatalyst was dispersed in a mixed solution of Na_2_S and Na_2_SO_3_ as a sacrificial agents under visible light.	16.34 µmol for 3 h	[154]
F-TiO_2_/CdS-DETA, Pt as a cocatalyst	50 mg of the photocatalyst was dispersed in 100 mL of mixed aqueous solution containing 0.35 mg/L Na_2_S and 0.25 mg/L Na_2_SO_3_ with the use of Pt as a cocatalyst.	5342.86 µmol/h/g	[155]
CdS@TiO_2_@Au	20 mg of the photocatalyst was dispersed in 40 ml of aqueous solution containing 0.1 M Na_2_S and 0.1 M Na_2_SO_3_ as the sacrificial agents under visible light.	1720 µmol/h/g	[156]
TiO_2_-Au-CdS	0.1 g of the sample was immersed in an aqueous solution containing 0.1 M Na_2_S and 0.1 M Na_2_SO_3_ as the sacrificial agents under visible light.	1810 µmol/h/g	[157]
*N*-TiO_2_/g-C_3_N_4_@Ni_x_P	50 mg photocatalyst was suspended in a 100 mL solution containing 10 vol.% triethanolamine (TEOA) under 300 W Xe lamp irradiation.	5438 µmol/h/g	[158]
TiO_2_/CdS/CNT	0.1 g of photocatalyst was dispersed in solution containing 70 mL of distilled water or seawater and 30 mL of sacrificial agent. The photocatalyst were irradiated using three visible-light sunlamps of each 100 W and UV lamp of 8 W.	3502 µmol/h from pure water and 1373 µmol/h from seawater	[159]
WS_2_/ C-TiO_2_/ g-C_3_N_4_	50 mg of photocatalyst was added in to 80 mL TEOA aqueous solution with the use of Pt as a cocatalyst.	17726 for DI water and 29978 µmol/g for seawater	[148]
TiO_2_/La_2_O_2_CO_3_/rGO	0.05 g of powder catalyst was dispersed in 80 Ml ethylene-glycol water (5/95, v/v) solution under UV-vis light.	583 µmol/h	[160]
TiO_2_-Cu@C	Photocatalyst was dispersed in methanol aqueous solution under UV-vis light.	3911 µmol/g/h	[161]
